# LAB Fermentation Improves Production of Bioactive Compounds and Antioxidant Activity of *Withania somnifera* Extract and Its Metabolic Signatures as Revealed by LC-MS/MS

**DOI:** 10.4014/jmb.2111.11018

**Published:** 2022-01-21

**Authors:** Jinhui Yu, Yun Geng, Han Xia, Deyuan Ma, Chao Liu, Rina Wu, Junrui Wu, Shengbo You, Yuping Bi

**Affiliations:** 1 Institute of Crop Germplasm Resources, Shandong Academy of Agricultural Sciences, Jinan 250100, P.R. China; 2 College of Life Science, Shandong Normal University, Jinan 250100, P.R. China; 3 College of Food Science, Shenyang Agricultural University, Shenyang 110866, P.R. China

**Keywords:** *Withania somnifera* extract, lactic acid bacteria, fermentation, antioxidant activity, metabolic signatures

## Abstract

In this study we investigated the effect of lactic acid bacteria (LAB) fermentation on the ingredients and anti-oxidant activity of *Withania somnifera* extract. Four strains of LAB could proliferate normally in medium containing *W. somnifera* extract after the pH reached 3.1~3.5. LAB fermentation increased the content of alcohols and ketones, endowing the extract with the characteristic aroma of fermentation. Compared to the control, the DPPH and ABTS free radical scavenging rates in the fermented samples were significantly improved, ranging from 48.5% to 59.6% and 1.2% to 6.4%. The content of total phenols was significantly increased by 36.1% during the fermentation of mixed bacteria. Moreover, the original composition spectrum of the extract was significantly changed while the differentially accumulated metabolites (DAMs) were closely related to bile secretion, tryptophan metabolism and purine metabolism. Therefore, LAB fermentation can be used as a promising way to improve the flavor and bioactivity of the extracts of *W. somnifera*, making the ferments more attractive for use as functional food.

## Introduction

*Withania somnifera* is a plant naturally distributed in the dry regions of Asian countries such as India, Pakistan, and Afghanistan as well as South Africa [1]. Its roots are specifically used in medicinal and clinical applications that have a long history dating back to 5000 BCE to Hindu Ayurveda medical practices [2, 3]. Previous studies have shown that *W. somnifera* is notably rich in phenolic compounds, flavanoids, alkaloids and steroidal lactones [4, 5]. The therapeutic applications of *W. somnifera* are broad and include anticancer [4, 5], anti-inflammatory, sedative, hypnotic and narcotic, reproductive [7], and antiangiogenic effects [8], in addition to ischemic stroke prevention [9] and recovery from neurodegenerative disorders [10]. As health-benefiting herbaceous plants are becoming an essential ingredient in new foods, *W. somnifera* could also be a valuable material for the functional food industry.

Fermentation is an important form of plant-based processing. There is growing interest in innovative products based on plants fermented by probiotics. Manwar *et al*. compared the antioxidant capacity of a liquid polyherbal formulation prepared by different fermentation processes, and the results showed that the traditional formulation exhibited the highest activity [11]. Chérif Rabhi *et al*. used the fungus *Beauveria bassiana* ATCC 7159 to ferment the mixture of extracts of *W. somnifera*, *Emblica officinalis*, and *Bacopa monnieri*. The fermentation promoted the fluidization of the starting dense mixture, removed the glucogallin from *E. officinalis* extract and increased the gallic acid content. Moreover, as the fermented extract was free of any toxicity and showed antiangiogenic potency, it was developed as a nutraceutical antiangiogenic treatment for age-related macular degeneration and commercialized in an oral form named Ethnodyne-Visio [8]. Lactobacilli are among the most important probiotic bacteria as many studies have shown that fermentation with lactic acid bacteria (LAB) could improve the physicochemical properties, bioactive compound content or profile, as well as the antioxidant potential and/or sensory evaluation of plant substrates [12, 13]. However, scant research has been done on the LAB fermentation of the ingredients and activity changes of *W. somnifera*.

In this study, we investigated for the first time the effects of single- or mixed-strain fermentation using four LAB strains on the extract of *W. somnifera*. We evaluated parameters such as the dynamic changes during the fermentation, component analysis of the total flavonoid and polyphenol content, and in vitro antioxidant activity. Moreover, we also analyzed the flavor and the non-target metabolic products of *W. somnifera* extract before and after fermentation to provide theoretical reference and technical support for developing functional probiotic drinks.

## Materials and Methods

The *W. somnifera* extract (aqueous extract, 20:1) was purchased from Sanyuan Longsheng Biotechnology Co.(China). Peptone, beef extract and yeast extract were obtained from Aoboxing Biotechnology Co., China. Glucose, monopotassium phosphate, Na-acetate, magnesium sulfate, ethanol, sodium nitrite, aluminum nitrate, ethyl acetate, and sodium hydroxide were purchased from Kermel Scientific Co. (China). Folin–Ciocalteu phenol reagent was obtained from Solarbio. In addition, 2,2-diphenyl-1-picrylhydrazyl (DPPH) and 2,6-Di-tert-butyl-4-methylphenol (BHT) were purchased from Sigma-Aldrich Inc. (Germany). Pyrogallic acid, 2,2′-Azino-bis(3-ethylbenzothiazoline-6-sulfonic acid) diammonium salt, ascorbic acid and rutin were obtained from Aladin Industries Inc. (China).

### Microorganisms and Culture Conditions

*Lactiplantibacillus plantarun* DY-1, *Lacticaseibacillus casei* KDB-LC, *Lactobacillus acidophilus* KDB-03 and *Limosilactobacillus fermentum* KDB-08 were previously isolated from natural fermented samples and preserved at the China Center for Type Culture Collection (CCTCC), and the preservation numbers were CCTCCM2016137, CCTCCM2016431, CCTCCM2016429, and CCTCCM2016430. All strains were preserved in glycerol solution (30%, v/v) and maintained at -80°C. The strains were activated in de Man Rogosa and Sharpe (MRS) medium and cultured at 37°C for 24 h. The cells were harvested by centrifugation at 3,500 ×*g* for 10 min at 4°C using a Multifuge X3R centrifuge (Thermo scientific, USA). The cells were washed twice with sterilized water and suspended for further inoculation.

### Plant Medium and Fermentation Treatment

The extract medium contained 2% extracts of *W. somnifera* (aqueous extract (20:1)), 2% glucose and 1.25%peptone. The sterilization was processed at 115°C for 20 min. The prepared media were inoculated with either a single strain or mixture of the above strains (1:1:1:1) to a final concentration of 7.0 log CFU/ml and marked as F1, F2, F3, F4 and FMIX. The sterilized and unfermented treatment served as the control check (CK). All the cultures were incubated at 37°C. To compare the differences among the groups, the fermented and unfermented samples were collected at 0, 24, 48, and 72 h. After 72 h fermentation, the samples were centrifuged at 3,500 ×*g* for 10 min at 4°C. The supernatants were obtained and stored at -80°C for further analysis.

### Determination of Fermentation Parameters

The bacteria amounts were detected every 24 h during fermentation. Serial dilutions of the samples at different fermentation times were spotted on MRS agar plates. After 48 h growth, the colonies in the appropriate dilution (30~300 colonies in a 100 μl spot with a plate of 9.0 cm diameter) were counted.

The value of pH was determined by a FE20 pH analyzer (Mettler Toledo, USA). Total acid was measured according to PRC National Standard GB/T 12456-2008. The determination of reducing sugar referred to previous study [14].

### Assessment of Antioxidant Activities and Components

The DPPH and ABTS radical scavenging activity assay, FRAP value and total antixiodiant capacity were performed as described in previous literature [15, 16] except for BHT taken as reference.

The content of total polyphenols and total flavonoids (TPC and TFC) was determined according to previously described methods with some modifications [17]. Briefly for TPC, sample solutions (1.0 ml, diluted 5 times with distilled water) and Folin-Ciocalteu reagent (2.0 ml, diluted 10 times with distilled water) were mixed thoroughly, and 2 ml Na_2_CO_3_ (0.02 g/ml) was added 5 min later. Then, the absorbance of the mixtures was measured at 760 nm after 1 h. Results were expressed as milligrams of gallic acid equivalent per milliliter (mg GAE/ml). Briefly for TFC, sample solutions (1.0 ml) were mixed with 1.0 ml NaNO_2_ (0.05 g/ml) and 1.0 ml AlCl_3_ (0.1 g/ml). The mixture was incubated for 5 min, and combined with 10.0 ml NaOH (1 mol/l) in a 25-ml volumetric flask. After mixing, the absorbance was measured at 510 nm after 15 min. Results were expressed as milligrams of rutin equivalents per milliliter (mg RE/ml).

### Analysis of Volatile Components

Samples were analyzed by the same instruments and procedures with some modification [18]. Sample detection was conducted as follows: 1.0 g sample was incubated in the headspace at 50°C for 15 min. Then, 500 μl of headspace was automatically injected by means of a heated syringe (60°C) into the heated injector (80°C). Nitrogen was used as carrier and drift gas. The carrier gas was passed through a GC–IMS injector by inserting the sample into the GC column. The initial carrier gas flow was 2 ml/min for the first 2 min and then was increased to 100 ml/min until 20 min. The ions were placed into a drift tube (9.8 cm length) through the shutter grid, which was operated at a constant voltage (500 Vcm^−1^) and temperature (45°C). The drift gas flow was set at a constant flow of 150 ml/min. Each spectrum had an average of 12 scans, a repetition rate of 30 ms, a grid pulse width of 100 μs and a sampling frequency of 150 kHz.

### UPLC-TripleTOF/MS-Based Metabolomics

**Sample Preparation**

The metabolites (200 μl) were extracted using a 800 μl methanol:acetonitrile (1:1, v/v) solution with 0.02 mg/ml L-2-chlorophenylalanin as internal standard. The mixture was vortexed for 30 s, and sonicated at 40 kHz for 30 min at 5°C. The samples were placed at −20°C for 30 min to precipitate the proteins. After centrifugation at 13,000 ×*g* at 4°C for 15 min, the supernatants were carefully transferred to sample vials for LC-MS/MS analysis. The pooled quality control (QC) sample was prepared by mixing equal volumes of all the samples.

### Acquisition of LC-MS/MS Data

Chromatographic separation of the metabolites was performed on an ExionLC AD system (AB Sciex, USA) equipped with an ACQUITY UPLC HSS T3 column (100 mm × 2.1 mm i.d., 1.8 μm; Waters, USA). The mobile phases consisted of 0.1% formic acid in water:acetonitrile (95:5, v/v) (solvent A) and 0.1% formic acid in acetonitrile:isopropanol:water (47.5:47.5:5, v/v/v) (solvent B). The solvent gradient changed according to the following condition: from 0 to 0.5 min, 0% B to 0% B; from 0.5 to 2.5 min, 0% B to 25% B; from 2.5 to 9 min, 25% B to 100% B; from 9 to 13 min, 100% B to 100% B; from 13 to 13.1 min, 100% B to 0% B, from 13.1 to 16 min, and 0%B to 5% B for equilibrating the systems. The sample injection volume was 10 μl and the flow rate was set to 0.4 ml/min. The column temperature was maintained at 40°C.

The UPLC system was coupled to a quadrupole time-of-flight mass spectrometer (TripleTOF 5600+; AB Sciex, USA) equipped with an electrospray ionization (ESI) source operating in positive and negative modes. The optimal conditions were set as follows: source temperature, 550°C; curtain gas, 30 psi; ion sources GS1 and GS2, both 50 psi; ion spray voltage floating (ISVF), −4,000 V in negative mode and 5000 V in positive mode; declustering potential, 80 V; and rolling collision energy (CE), 20–60 V for MS/MS analysis; cycle time, 510 ms. Data acquisition was performed in the Data-Dependent Acquisition (DDA) mode. The detection was carried out over a mass range of 50–1,000 m/z.

### Statistical Analysis

All the experiments were repeated three times. The data values were reported as the mean ± SD. The significance between groups was calculated with one-way ANOVA and Duncan's t-test. p-values less than 0.05 were considered statistically significant. Differential metabolites between two groups were summarized. Statistically significant among groups were selected with VIP value with different ranges and p-value less than 0.05.

## Results

### Dynamic Situation of Microbial Viability, pH Change, Residual Sugar and Total Acid During Fermentation

**Fig. 1:**
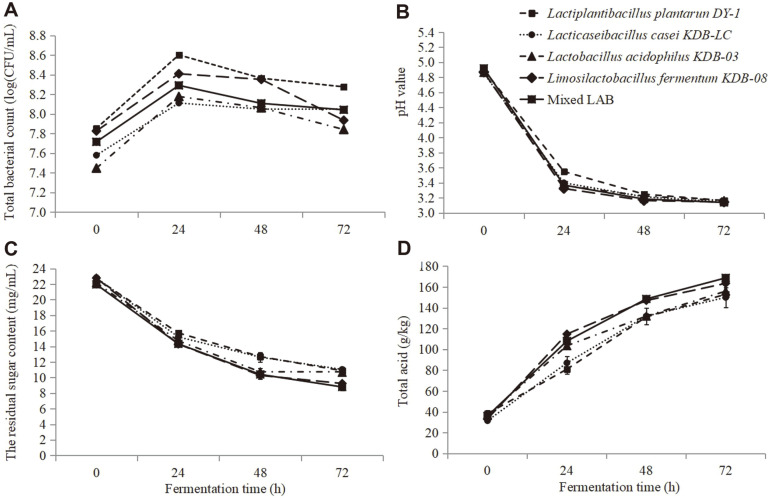
Changes in bacterial count, pH, residual sugar content and total acid content during the fermentation process. **A**, bacterial count; **B**, pH value; **C**, residual sugar content; **D**, total acid content.

The total bacterial amount of LAB was counted during different stages of the fermentation (Fig. 1A). The inoculation concentration was 7.4~7.8 log CFU/ml, and the total amount reached to the highest level at 24 h (8.1~8.6 log CFU/ml), followed by a decrease to 7.8~8.2 log CFU/ml at the end of the fermentation. The cell counts of the mixed bacteria stayed at an intermediate level, and maintained at 8.0 at 72 h fermentation. Normal growth of the strains present in the medium indicated that extracts of *W. somnifera* at the concentration of 40 mg/ml had no inhibitory effect on the growth and proliferation of cells.

The pH reduction rate was in agreement with the LAB count after fermentation. The pH value of single- or mixed-bacteria fermentation decreased sharply during fermentation to pH 3.3~3.5 within 24 h, whereas the fermentation by DY-1 was slightly slower. The pH values of these samples declined slowly for another 48 h and finally reached to pH 3.1 (Fig. 1B), which was much more effective than the household fermentation of carrot juice [19].

The residual sugar content was significantly decreased as the viable cell count proliferated, while the decline tended to slow down after 24 h and the residual sugar content remained at 8.7~11 mg/ml until 72 h later (Fig. 1C). Acidity significantly increased during the first 24 h fermentation. As the fermentation progressed, acidity tended to increase slightly in all samples. Final acidity values were 150.0~168.7 g/kg (Fig. 1D). The increase in acidity during the fermentation period could be due to the lactic acid or other organic acids produced by LABs [20].

### Effect of Fermentation on Antioxidant Activity

**Fig. 2:**
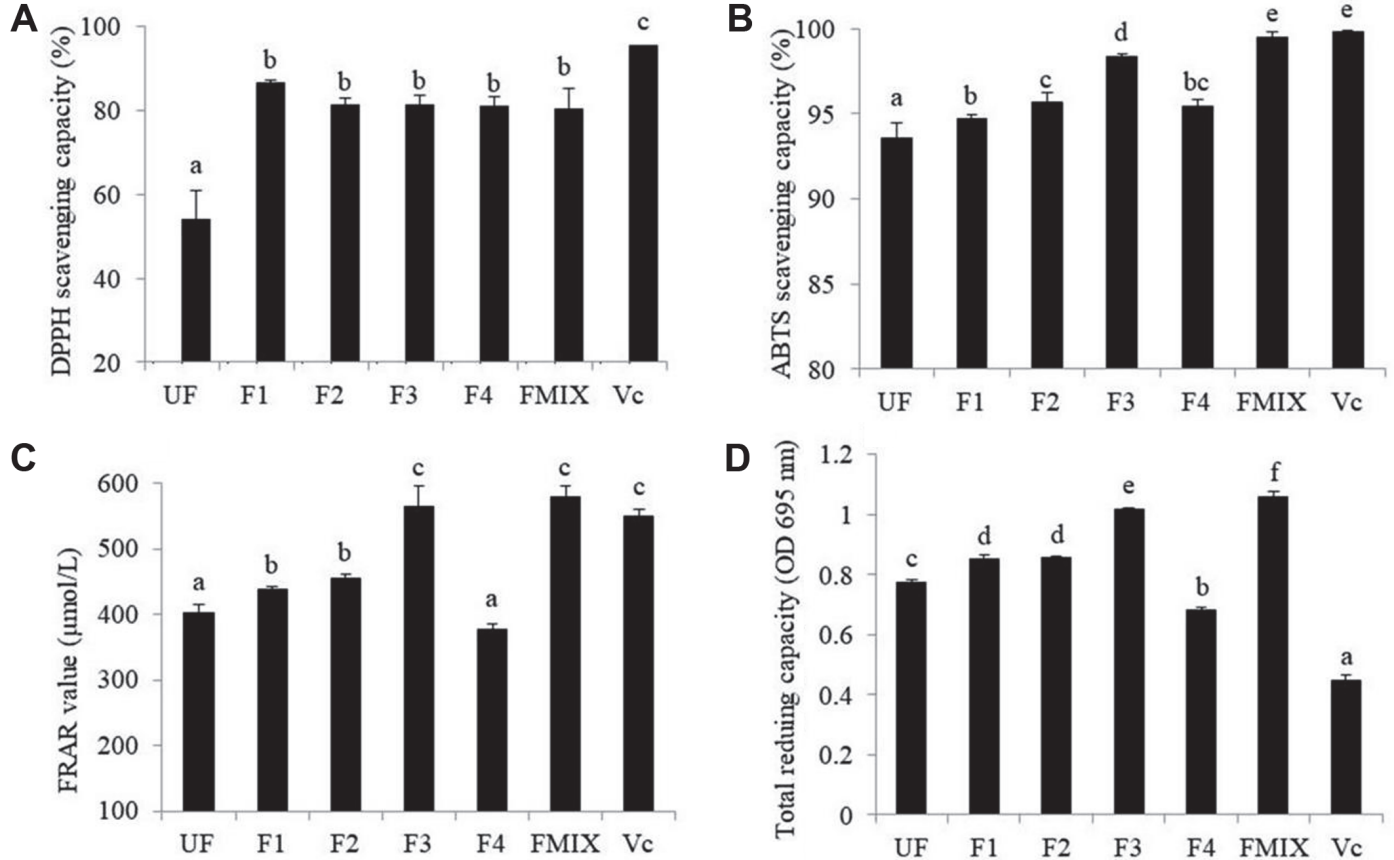
Antioxidant activity of the extract of *W. somnifera* before and after fermentation. **A**, DPPH scavenging capacity; **B**, ferrous chelating activity; **C**, total reducing capacity; **D**, FRAR value. Fermented supernatant was diluted 5 times and then used to measure. F1, fermented by *Lactiplantibacillus plantarun* DY-1; F2, fermented by *Lacticaseibacillus casei* KDBLC; F3, fermented by *Lactobacillus acidophilus* KDB-03; F4 fermented by *Limosilactobacillus fermentum* KDB-08; FMIX, fermented by mixture of the above strains. Different letters indicate significant differences (*p* < 0.05).

**Fig. 3:**
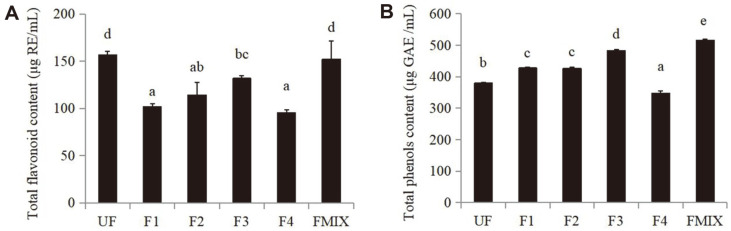
Changes of total flavonoid and total phenol content before and after the fermentation. **A**, TFC, total flavonoid content; **B**, TPC, total polyphenol content. F1, fermented by *Lactiplantibacillus plantarun* DY-1; F2, fermented by *Lacticaseibacillus casei* KDB-LC; F3, fermented by *Lactobacillus acidophilus* KDB-03; F4 fermented by *Limosilactobacillus fermentum* KDB-08; FMIX, fermented by mixture of the above strains. Different letters indicate significant differences (*p* < 0.05).

The antioxidant capacities were determined by the DPPH and ABTS free radical scavenging capacity, FRAP, and total antixiodiant capacity (Fig. 2). Compared to the control, the DPPH and ABTS free radical scavenging rates in the fermented samples were significantly improved, and ranged from 48.5 ± 9.13% to 59.6 ± 1.74% and 1.21 ± 0.29% to 6.39 ± 0.27% (Figs. 2A and 2B). The FRAP and total antioxidant capacity in the fermented samples were also increased except F4 (Figs. 2C and 2D). There were obvious differences in the ABTS free radical scavenging rate, FRAR and total antioxidant capacity among the single and mixed fermentations (*p* > 0.05), suggesting that the different strains or combinations of strains will significantly affect the fermentation effect. As flavonoids and phenols are mainly antioxidant components, the content of total flavonoids and phenols was determined. The results showed that total flavonoid content significantly decreased by single fermentation (*p* > 0.05), while total phenol content significantly increased (*p* > 0.05) with the maximum rise up to 36.1% by the fermentation of mixed bacteria (Figs. 3A and 3B).

### Analysis of Volatile Components in Fermented and Unfermented Samples

**Fig. 4:**
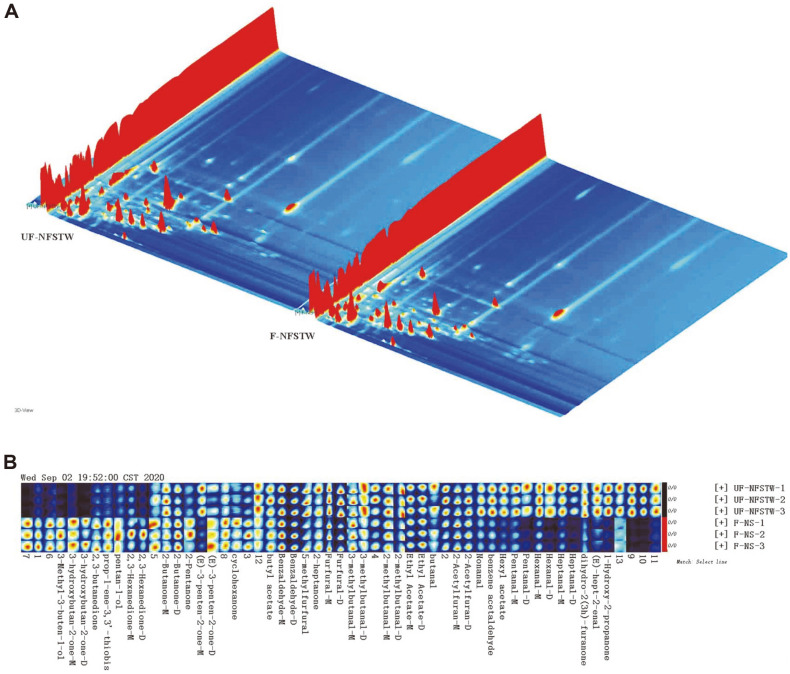
3D-topographic of GC-IMS analysis and gallery plot (finger-print) of volatile compounds of the fermented or unfermented samples. (**A**) 3D-topographic of GC-IMS analysis, the color of the signal peak represents the concentration of the substance, among which white indicates a lower concentration and red indicates a higher concentration, and the darker the color, the higher the concentration; (**B**) Gallery plot (finger-print) of volatile compounds, row represents the volatile composition of a sample and column represents the signal peaks of a volatile substance in different samples. UFNFSTW, *W. somnifera* extract with sterilized and unfermented treatment; F-NS, *W. somnifera* extract with sterilized and fermented by the mixture of LAB.

Forty-two identified and thirteen unidentified volatile components were detected in the samples, and fermentation significantly changed the abundance spectra of the volatile components when compared to the unfermented extract of *W. somnifera* (Fig. 4).

### The main flavored components of *W. somnifera* extract included alcohols, aldehydes, esters, ketones and furan (Table S1). Most of the aldehyde components were decreased and others were increased after fermentation as shown by the relative contents (Table 1). The measured components could be divided into three categories. The first category involved components of higher content in unfermented sample, which included 1-hydroxy-2-propanone, (E)-hept-2-enal, dihydro-2(3h)-furanone, heptanal, hexanal, pentanal, (E)-3-penten-2-one-M, 2-butanone-M, hexyl acetate, 5-methylfurfural, benzene acetaldehyde and nonanal. The second category involved components that were significantly increased after fermentation, which included cyclohexanone, (E)-3-penten-2-one-D, 2-pentanone, 2-butanone-D, 2,3-hexanedione, pentan-1-ol, prop-1-ene-3,3'-thiobis, 2,3-butanedione,

3-hydroxybutan-2-one and 3-methyl-3-buten-1-ol. The third group of components consisted of those not differing between fermented and unfermented samples, and included 2-acetylfuran, butanal, ethyl acetate, 3-methylbutanal, furfural, 2-heptanone, benzaldehyde and butyl acetate.

### Effect of Fermentation on Untargeted Substances Based on Metabonomics

**Fig. 5:**
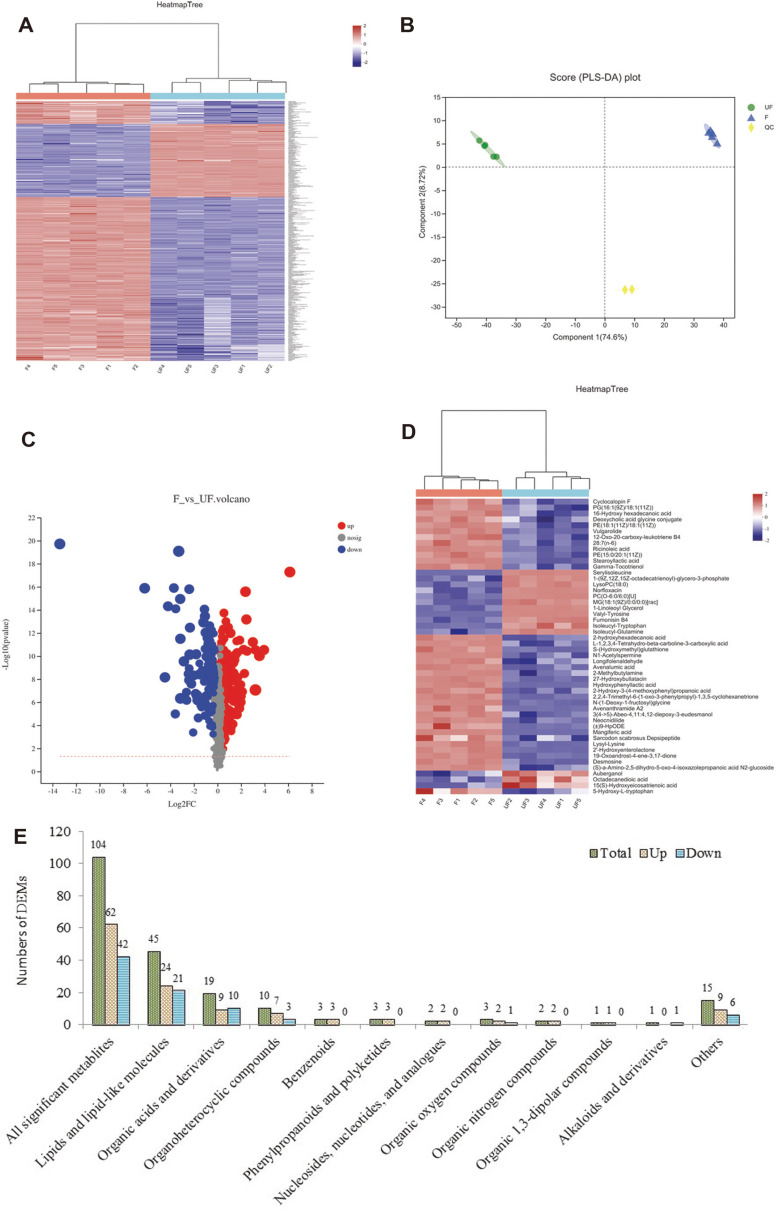
Overview of widely targeted metabolome analysis of fermented and unfermented extracts of *W. somnifera*. (**A**) Heatmap visualization of metabolites. Red indicates high abundance, whereas low relative metabolites are shown in green. (**B**) PLS-DA analysis of metabolites. (**C**) Volcano plot of metabolites. (**D**) Heatmap of DAMs. (E) Category and number of DAMs.

A widely targeted metabolome analysis was performed on fermented and unfermented samples, that is, F and UF. A total of 398 metabolites were obtained in all samples, as presented in the heatmap visualization (Fig. 5A, Table S2), showing the distinct hierarchical clustering of samples by fermentation.

In the PCA plot (Fig. 5B), the QC samples were grouped together, indicating that the QC samples had similar metabolic profiles and that the entire analysis was stable and repeatable. As the fold-change threshold was set to greater than one, a total of 104 differentially accumulated metabolites (DAMs) were obtained for the comparison of F versus UF, of which 62 metabolites were upregulated and 42 metabolites were downregulated in F (Figs. 5C and 5E, Table S3). A heatmap of DAMs confirmed the significant differences in the metabolome of F and UF (Fig. 5D). The detected compounds in the samples mainly belonged to the following classes (Fig. 5E): (1) lipids and lipid-like molecules, (2) organic acids and derivatives, (3) organoheterocyclic compounds, (4) phenylpropanoids and polyketides, (5) organic oxygen compounds, (6) benzenoids, (7) organic nitrogen compounds, (8) nucleosides, nucleotides and analogues, (9) hydrocarbons, (10) alkaloids and derivatives, (11) lignans, neolignans and related compounds, (12) organic 1,3-dipolar compounds, (13) organosulfur compounds, (14) others.

**Fig. 6:**
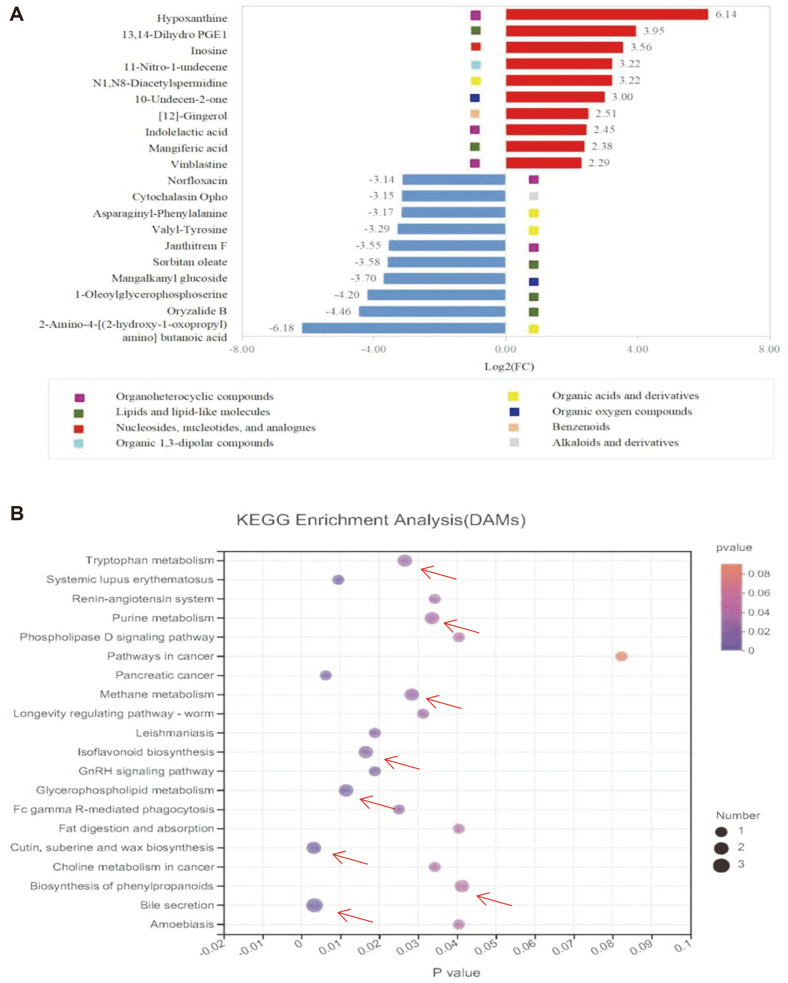
The top 10 regulated DAMs and KEGG enrichment pathway of DAMs. (**A**) Top 10 regulated DAMs. Red bars indicate upregulated DAMs. Blue bars indicate downregulated DAMs. (**B**) KEGG enrichment pathway of DAMs. The arrows highlight the significant enrichment metabolism pathways.

The top 10 upregulated and downregulated DAMs were screened out (Fig. 6A), of which three, hypoxanthine, 13,14-dihydro PGE1, and inosine, were the most upregulated DAMs while three others, 2-amino-4-[(2-hydroxy-1-oxopropyl) amino] butanoic acid, oryzalide B, and 1-oleoylglycerophosphoserine were the most downregulated DAMs. KEGG pathway analysis showed that the DAMs were mainly enriched in the eight pathways of bile secretion, tryptophan metabolism, purine metabolism, methane metabolism, isoflavonoid biosynthesis, glycerophospholipid metabolism, cutin, suberine and wax biosynthesis, and biosynthesis of phenylpropanoids (Fig. 6B).

## Discussion

The *W. somnifera* extract has good physiological activity as it comes from a herbaceous plant with adaptogenic properties [3]; however, the fermentation by LAB has not been reported yet. Therefore, we carried out the study of *W. somnifera* extract fermentation by LAB. Strains were isolated, screened and saved in our laboratory. The strains all exhibit strong resistance to artificial gastric juice and artificial intestinal juice while also having high antioxidant activity and cholesterol scavenging ability (data not shown). Products based on these strains have been developed and received good feedback from consumers for more than ten years. To develop the potential applications of these strains, we have been conducting studies related to plant-based fermentation over the past few years. Previous results had suggested that these strains could improve nutritional characteristics and increase the content of functional components and bioactivities [18]. The results of this article showed that the total number of LAB increased in the medium containing *W. somnifera* extracts, and the residual sugar and the pH declined in the fermented liquid, while total acid content increased, indicating that LAB could grow and proliferate normally in culture medium containing the extract of *W. somnifera*. Compared to fermentation by single strain, the mixed treatment showed the highest glucose consumption rate and total acid content, which might be related to the synergistic utilization of different metabolic pathways of mixed cultures [21].

Flavor plays an important role in food quality, while fermentation is one of the best ways to improve the flavor of food [22]. LAB strains are commonly used for fermentation in the food industry. LAB fermentation increases ketones, acids, alcohols, aldehydes, esters, and aromatic hydrocarbons in milk. Among these, some key flavor volatile compounds have been identified, including acetaldehyde, 3-methylbutanal, acetoin, 2-heptanone, acetic acid, butanoic acid, and 3-methyl-1-butanol [23]. LAB fermentation also significantly improves the taste and flavor of fruits and vegetables, while the main flavor substances produced by different strains are different. Some examples include *L. casei* and *L. rhamnosus* (Type A FVFs), which contribute to umami taste, and *L. plantarum* and *Lactobacillus acidophilus* (Type B FVFs), which contribute to sour taste. *Limosilactobacillus fermentum* might be a potential critical contributor to the production of volatile compounds [24]. In this article, a mixture fermentation of *L. plantarum* DY-1, *L. casei* KDB-LC, *L. acidophilus* KDB-03 and *L. fermentum* KDB-08 endowed *W. somnifera* extract with the characteristic aroma of fermentation and increased the content of alcohols and ketones, which might contribute to improving the flavor of the fermented *W. somnifera* extract. Moreover, the content of aldehydes was decreased, which might help to develop a better overall flavor because aldehydes can lead to off-flavors at high concentration [25].

Oxidative stress is the main culprit leading to a variety of diseases, while plant components play an important antioxidant role [26]. In this article, the antioxidant activity of the samples before and after fermentation was determined, and the results showed that the DPPH and ABTS free radical scavenging ability, total antioxidant capacity and FRAR value of the samples were significantly increased after fermentation (except for the total antioxidant capacity and FRAR value of F4), which was closely related to the changes of their components. Phenolics exert health benefits in humans and the metabolism of phenolics might contribute to bacterial stress response when microorganisms are in hostile conditions [27]. LAB were able to increase the concentration of phenolic compounds, metabolize phenolic acids, and convert flavonoid glycosides and tannins [28]. We found that the content of total flavonoids in *W. somnifera* extract decreased and the content of total phenols increased overall after the single-LAB strain fermentation, while the content of total flavonoids was not significantly changed after the mixed-LAB strain fermentation (Fig. 4A). The content of total phenols significantly increased by 36.08% (Fig. 4B), and this was consistent with previous studies [29].

Food processing has drastic effects on the chemical composition of foods [30]. Due to the complexity of the fermentation process, as well as the non-determinacy of the extract, the changes in total phenols and total flavonoids could not fully reflect the fermentation effect of *W. somnifera* extracts. Untargeted mass spectrometry analysis of food samples has the potential to increase our chemical understanding of these processes by detecting a broad spectrum of chemicals, and was widely used in the analysis of fermentation products [30]. One hundred and four DAMs were detected after fermentation treatment by this investigation, mainly belonging to lipids and lipid-like molecules, organic acids and derivatives, and organoheterocyclic compounds. The lipids and lipid-like molecules comprised a class of fatty acid esters, fatty acids and conjugates, fatty acyl glycosides, hydroxysteroids, diterpenoids, and glycerophosphocholines, and the organic acids and derivatives included amino acids, peptides, keto acids, and hydroxy acids, that changed in abundance after fermentation which might be produced via the metabolism of protein, carbohydrates, fats, and amino acids by LAB [31]. As one of the most upregulated DAMs, hypoxanthine was considered as a potential marker for assessing meat freshness and clinical diagnosis [32]. However, inosine, formed by a β-glycosidic bond between hypoxanthine and ribose [33], became a novel microbiota-derived immunostimulatory metabolite [34]. The conversion of hypoxanthine to inosine might be closely related to the fermentation process, thus affecting the accumulation of substances. Among the other upregulated DAMs, 13,14-dihydro PGE1 could significantly inhibit the stress-induced increase in mitotic activity [35]. Moreover, the KEGG enrichment pathway of DAMs pointed to bile secretion, tryptophan metabolism, and purine metabolism, which suggested that the fermented extract might play a role in the diseases involved in these metabolism-related pathways. However, further studies are still needed to clarify the effect of these fermentation parameters on the accumulation of functional substances, and to verify the potential activities of the fermented product and reveal related mechanisms.

In conclusion, *L. plantarum* DY-1, *L. casei* KDB-LC, *L. acidophilus* KDB-03 and *L. fermentum* KDB-08 grew well in the medium with *W. somnifera* extracts and LAB fermentation significantly increased the antioxidant activity and flavor components of the *W. somnifera* extracts. Under the metabolic action of the mixed bacteria, the original composition spectrum of the extract was significantly changed, and the DAMs were closely related to bile secretion, tryptophan metabolism and purine metabolism. Therefore, LAB fermentation can be used as a promising way to improve the flavor and bioactivity of the extracts of *W. somnifera*, making the ferments more attractive for use as functional food.

## Supplemental Materials

Supplementary data for this paper are available on-line only at http://jmb.or.kr.

## Figures and Tables

**Table 1:** Peak intensity and relative contents of volatile compounds in unfermented and fermented samples.

Compound	Peak intensity		Relative content (%)	
	
	Unfermented	Fermented	Unfermented	Fermented
Alcohol				
Nonanal	894.12 ± 67.02	581.01 ± 31.63	2.14 ± 0.14	1.69 ± 0.14
3-Methyl-3-buten-1-ol	49.45 ± 3.24	503.07 ± 66.52	0.12 ± 0.01	1.46 ± 0.16
Pentan-1-ol	50.64 ± 4.73	108.52 ± 15.43	0.12 ± 0.01	0.31 ± 0.04
Aldehyde				
Furfural-D	8719.76 ± 201.28	4587.56 ± 746.40	20.85 ± 0.80	13.25 ± 1.53
Furfural-M	4930.27 ± 85.12	3767.15 ± 182.98	11.79 ± 0.38	10.92 ± 0.04
Benzaldehyde-D	1833.66 ± 86.96	1690.94 ± 205.50	4.38 ± 0.11	4.89 ± 0.36
Benzaldehyde-M	1763.98 ± 39.03	1775.85 ± 67.29	4.22 ± 0.01	5.15 ± 0.13
3-Methylbutanal-D	2130.97 ± 210.27	1581.02 ± 88.97	5.09 ± 0.39	4.58 ± 0.12
3-Methylbutanal-M	1025.37 ± 74.42	982.46 ± 52.78	2.45 ± 0.23	2.86 ± 0.31
2-Methylbutanal-D	1724.40 ± 217.78	1321.90 ± 70.68	4.11 ± 0.43	3.83 ± 0.09
2-Methylbutanal-M	445.19 ± 10.14	393.47 ± 15.42	1.06 ± 0.02	1.14 ± 0.08
5-Methylfurfural	984.55 ± 30.61	763.59 ± 85.36	2.35 ± 0.12	2.21 ± 0.17
Hexanal-D	575.40 ± 95.51	65.02 ± 17.01	1.37 ± 0.20	0.19 ± 0.04
Hexanal-M	802.56 ± 32.62	325.37 ± 48.18	1.92 ± 0.04	0.94 ± 0.09
Benzene acetaldehyde	333.80 ± 10.74	197.09 ± 22.08	0.80 ± 0.03	0.57 ± 0.05
Heptanal-D	129.95 ± 28.73	25.94 ± 5.48	0.31 ± 0.06	0.08 ± 0.01
Heptanal-M	660.51 ± 47.36	259.14 ± 33.62	1.58 ± 0.09	0.75 ± 0.06
Pentanal-D	222.08 ± 54.64	38.96 ± 14.54	0.53 ± 0.12	0.11 ± 0.04
Pentanal-M	698.04 ± 58.55	372.44 ± 41.02	1.67 ± 0.11	1.08 ± 0.07
Butanal	69.69 ± 17.30	58.29 ± 11.53	0.17 ± 0.04	0.17 ± 0.03
(E)-Hept-2-enal	70.48 ± 1.28	28.37 ± 0.62	0.17 ± 0.00	0.08 ± 0.00
Ester				
Ethyl Acetate-D	1420.71 ± 163.31	1335.34 ± 57.44	3.40 ± 0.47	3.88 ± 0.29
Ethyl Acetate-M	884.31 ± 64.65	727.40 ± 26.66	2.12 ± 0.20	2.12 ± 0.19
Hexyl acetate	265.58 ± 24.34	115.67 ± 15.20	0.63 ± 0.05	0.34 ± 0.04
Butyl acetate	68.52 ± 3.38	72.24 ± 3.16	0.16 ± 0.01	0.21 ± 0.01
Prop-1-ene-3,3'-thiobis	23.80 ± 4.27	53.17 ± 3.72	0.06 ± 0.01	0.15 ± 0.01
Furan				
2-Acetylfuran-M	460.90 ± 9.69	486.20 ± 5.29	1.10 ± 0.05	1.41 ± 0.08
2-Acetylfuran-D	63.78 ± 6.76	51.89 ± 1.58	0.15 ± 0.02	0.15 ± 0.01
Ketone				
2-Butanone-D	2893.09 ± 468.10	3921.85 ± 190.44	6.90 ± 0.98	11.37 ± 0.22
2-Butanone-M	1046.35 ± 27.63	918.59 ± 42.54	2.50 ± 0.08	2.67 ± 0.23
Dihydro-2(3h)-furanone	1506.78 ± 80.05	748.71 ± 84.74	3.60 ± 0.24	2.17 ± 0.14
2,3-Bbutanedione	296.02 ± 9.19	752.69 ± 117.86	0.71 ± 0.04	2.19 ± 0.39
3-Hydroxybutan-2-one-D	32.34 ± 2.67	421.63 ± 94.41	0.08 ± 0.00	1.23 ± 0.30
3-Hydroxybutan-2-one-M	91.83 ± 4.52	492.66 ± 41.51	0.22 ± 0.01	1.43 ± 0.17
2,3-Hexanedione-D	82.55 ± 7.76	357.00 ± 159.54	0.20 ± 0.01	1.03 ± 0.43
2,3-Hexanedione-M	174.87 ± 8.32	337.74 ± 83.01	0.42 ± 0.01	0.98 ± 0.22
(E)-3-Penten-2-one-D	88.82 ± 25.11	120.44 ± 13.35	0.21 ± 0.06	0.35 ± 0.04
(E)-3-penten-2-one-M	260.21 ± 34.33	87.84 ± 7.17	0.62 ± 0.07	0.25 ± 0.01
2-Heptanone	124.37 ± 22.54	118.49 ± 9.27	0.30 ± 0.05	0.34 ± 0.02
2-Pentanone	62.64 ± 2.48	109.30 ± 26.33	0.15 ± 0.01	0.32 ± 0.08
1-Hydroxy-2-propanone	285.83 ± 9.98	64.33 ± 3.85	0.68 ± 0.02	0.19 ± 0.01
Cyclohexanone	20.67 ± 1.49	29.20 ± 6.63	0.05 ± 0.00	0.09 ± 0.02
Unknown compound				
1	220.53 ± 25.13	679.66 ± 75.69	0.53 ± 0.08	1.97 ± 0.15
2	832.71 ± 64.72	673.93 ± 56.01	1.99 ± 0.19	1.96 ± 0.21
3	28.56 ± 3.33	517.01 ± 11.96	0.07 ± 0.01	1.50 ± 0.09
4	189.83 ± 14.43	500.49 ± 31.27	0.45 ± 0.03	1.46 ± 0.17
5	313.03 ± 8.37	476.64 ± 28.17	0.75 ± 0.03	1.38 ± 0.04
6	471.24 ± 84.37	361.54 ± 75.83	1.13 ± 0.22	1.05 ± 0.24
7	407.81 ± 14.45	318.50 ± 11.93	0.97 ± 0.04	0.93 ± 0.08
8	30.61 ± 5.68	50.30 ± 7.16	0.07 ± 0.02	0.15 ± 0.03
9	41.44 ± 2.75	49.79 ± 5.37	0.10 ± 0.01	0.14 ± 0.02
10	580.49 ± 6.34	43.88 ± 5.08	1.39 ± 0.03	0.13 ± 0.01
11	327.52 ± 13.60	27.01 ± 4.98	0.78 ± 0.03	0.08 ± 0.01
12	70.81 ± 5.69	22.18 ± 2.10	0.17 ± 0.01	0.06 ± 0.00
13	62.50 ± 10.96	20.62 ± 1.33	0.15 ± 0.02	0.06 ± 0.01
