# Supplementary material for: LAB Fermentation Improves Production of Bioactive Compounds and Antioxidant Activity of Withania somnifera Extract and Its Metabolic Signatures as Revealed by LC-MS/MS
Source: J Microbiol Biotechnol. 2022 Jan 21;32(4):473–83. doi: 10.4014/jmb.2111.11018 (PMC9628816; doi:10.4014/jmb.2111.11018)
Supplement: Supplementary file 1 [file jmb-32-4-473-supple.pdf]

J. Microbiol. Biotechnol.
https://doi.org/10.4014/jmb.2111.11018

Supplementary Table 1.

The information of substances from GC-IMS of the different samples.

Count	Compound	Functional group	Odour descriptor	Formula	MW	Rt [sec]	Dt [a.u.]
U1	Nonanal	Alcohol	Intense smell of oil and sweet orange	$C_9H_{18}O$	142.2	506.104	1.4829
U2	Benzene acetaldehyde	Aldehyde	Hyacinthlike aroma	C_8H_8O	120.2	406.557	1.25801
U3	5-Methylfurfural	Aldehyde	Caramel and spicy aromas	$C_6H_6O_2$	110.1	322.381	1.12682
U4	Hexyl acetate	Ester	Pleasant fruity aromas and sweet pear acidity	$C_8H_{16}O_2$	144.2	356.764	1.41389
U5	Heptanal-M	Aldehyde	Fruity scent	$C_7H_{14}O$	114.2	263.733	1.332
U6	Furfural-M	Aldehyde	Like almonds	$C_5H_4O_2$	96.1	223.322	1.08313

U7	Furfural-D	Aldehyde	Like almonds	$C_5H_4O_2$	96.1	223.322	1.32961
U8	Hexanal-M	Aldehyde	Aromas of grease, grass and apple	$C_6H_{12}O$	100.2	203.382	1.26021
U9	Hexanal-D	Aldehyde	Aromas of grease, grass and apple	$C_6H_{12}O$	100.2	202.85	1.55933
U20	(E)-3-Penten-2-one-M	Amines	Fruity and pungent	C_5H_8O	84.1	179.426	1.09095
U26	Pentanal-M	Aldehyde	Special fragrance	$C_5H_{10}O$	86.1	162.124	1.1894
U28	2-Methylbutanal-D	Aldehyde	Unique aroma of cocoa, coffee and malt	$C_5H_{10}O$	86.1	152.041	1.38944
U29	3-Methylbutanal-D	Aldehyde	Apple aromas	$C_5H_{10}O$	86.1	148.179	1.41053
U30	Ethyl Acetate-M	Ester	Strong ethereal smell	$C_4H_8O_2$	88.1	138.138	1.09521

U37	1-Hydroxy-2-propanone	Ketone	Fragrance	$C_3H_6O_2$	74.1	160.344	1.23034
U38	Pentanal-D	Aldehyde	Special fragrance	$C_5H_{10}O$	86.1	161.889	1.41819
U40	Butanal	Aldehyde	Choking odor	C_4H_8O	72.1	122.883	1.28114
U49	2-Acetylfuran-D	Furan	Aromas of almond, nut, yeast, milk and sweet caramel	$C_6H_6O_2$	110.1	271.932	1.43718
U50	(E)-Hept-2-enal	Aldehyde	Grass aroma and pungent odour	$C_7H_{12}O$	112.2	307.31	1.25412
U55	Dihydro-2(3h)-furanone	Ketone	Slight odour	$C_4H_6O_2$	86.1	270.923	1.08438
U&F4	Benzaldehyde-M	Aldehyde	Distinctive almond smell	C_7H_6O	106.1	312.866	1.15119
U&F 5	Benzaldehyde -D	Aldehyde	Distinctive almond smell	C_7H_6O	106.1	312.866	1.47541
U&F8	2-Acetylfuran-M	Furan	Aromas of	$C_6H_6O_2$	110.1	273.28	1.11574

			almond, nutty, yeast, milk and sweet caramel			1	
U&F10	2-Heptanone	Ketone	Fruit aroma similar to pear	$C_7H_{14}O$	114.2	256.289	1.26021
U&F31	Ethyl Acetate-D	Ester	Strong ethereal smell	$C_4H_8O_2$	88.1	137.173	1.33865
U&F46	Butyl acetate	Ester	Intense fruity aroma	$C_6H_{12}O_2$	116.2	209.891	1.23636
F15	2,3-Hexanedione-M	Ketone	Creamy aroma	$C_6H_{10}O_2$	114.1	199.66	1.0915
F16	2,3-Hexanedione-D	Ketone	Creamy aroma	$C_6H_{10}O_2$	114.1	196.996	1.35349
F24	3-Methyl-3-buten-1-ol	Alcohol	Nonpungent odor	$C_5H_{10}O$	86.1	177.146	1.24683
F25	3-Hydroxybutan-2-one-D	Ketone	Buttery odor	$C_4H_8O_2$	88.1	167.757	1.32771
F27	3-Methylbutanal-M	Aldehyde	Apple fragrance	$C_5H_{10}O$	86.1	147.986	1.17955

F32	2-Butanone -D	Ketone	Slightly pungent smell	C_4H_8O	72.1	130.414	1.24472
F33	2,3-Butanedione	Ketone	Special butter oil fragrance	$C_4H_6O_2$	86.1	126.359	1.17955
F34	2-Pentanone	Ketone	Pungent smell	$C_5H_{10}O$	86.1	158.799	1.11438
F35	3-Hydroxybutan-2-one-M	Ketone	Pleasant creamy aroma	$C_4H_8O_2$	88.1	169.999	1.06166
F36	(E)-3-Penten-2-one-D	Ketone	Fruity and pungent	C_5H_8O	84.1	179.075	1.34535
F39	2-Methylbutanal-M	Aldehyde	Unique aroma of cocoa, coffee and malt	$C_5H_{10}O$	86.1	154.165	1.1623
F41	2-Butanone-M	Ketone	Intense sweet taste	C_4H_8O	72.1	129.642	1.05879
F42	Heptanal-D	Aldehyde	Fruity	$C_7H_{14}O$	114.2	263.196	1.69506
F44	Prop-1-ene-3,3'-thiobis	Ethers	Smell of garlic	$C_6H_{10}S$	114.2	242.889	1.12304

F45	Cyclohexanone	Ketone	Earthy fragrance	$C_6H_{10}O$	98.1	258.683	1.15137
F53	Pentan-1-ol	Alcohol	Pleasant odor	$C_5H_{12}O$	88.1	190.439	1.25025

Supplemental Table 2.

The information of total 398 metabolites.

Number	Metabolite	Formula	FC(F/UF)	HMDB Superclass
1	Glycerophosphocholine	$C_8H_{20}NO_6P$	0.77	Lipids and lipid-like molecules
2	Thyroxine sulfate	$C_{15}H_{11}I_4NO_7S$	1.22	Organic acids and derivatives
3	Dihydrodeoxy-8-epiaustdiol	$C_{12}H_{14}O_4$	2.82	Organoheterocyclic compounds
4	Niazidin	$C_{15}H_{18}N_2O_6S$	1.27	-
5	Lapathinol	$C_{17}H1_6O_6$	1.29	-
6	Pentadecanol	$C_{15}H_{32}O$	1.88	Lipids and lipid-like molecules
7	19-Nor-5-androstenediol	$C_{18}H_{28}O_2$	2.60	Lipids and lipid-like molecules
8	Neoraucarpanol	$C_{22}H_{22}O_6$	1.66	-

9	4,8 Dimethylnonanoyl carnitine	$C_{18}H_{35}NO_4$	1.48	Lipids and lipid-like molecules
10	PA(16:0/18:2(9Z,12Z))	$C_{37}H_{69}O_8P$	2.75	Lipids and lipid-like molecules
11	2-Hydroxy-3-(4-methoxyphenyl)propanoic acid	$C_{10}H_{12}O_4$	1.70	Phenylpropanoids and polyketides
12	PGP(a-13:0/a-13:0)	$C_{32}H_{64}O_{13}P_2$	0.20	Lipids and lipid-like molecules
13	Trans-2-Hexenyl 2-methylbutyrate	$C_{11}H_{20}O_2$	2.47	Lipids and lipid-like molecules
14	2,2,7,7-Tetramethyl-1,6-dioxaspiro[4.4]non-3-ene	$C_{11}H_{18}O_2$	1.96	Organic oxygen compounds
15	Gamma-Glutamyltryptophan	$C_{16}H_{19}N_3O_5$	0.84	Organic acids and derivatives
16	PS(18:0/20:3(8Z,11Z,14Z))	$C_{44}H_{80}NO_{10}P$	2.88	Lipids and lipid-like molecules

17	Tanacetol A	$C_{17}H_{26}O_4$	1.72	Lipids and lipid-like molecules
18	Doxercalciferol	$C_{28}H_{44}O_2$	3.58	-
19	Isopentyl gentiobioside	$C_{17}H_{32}O_{11}$	2.82	Lipids and lipid-like molecules
20	Porphobilinogen	$C_{10}H_{14}N_2O_4$	2.56	Organic nitrogen compounds
21	Hexadecanedioic acid mono-L-carnitine ester	$C_{23}H_{43}NO_6$	1.24	Lipids and lipid-like molecules
22	(5Z,8Z)-1,5,8-Heptadecatriene	$C_{17}H_{30}$	1.43	Hydrocarbons
23	1,2-Anhydridoniveusin	$C_{20}H_{24}O_7$	1.54	Lipids and lipid-like molecules
24	Taraxinic acid glucosyl ester	$C_{21}H_{28}O_9$	1.61	Lipids and lipid-like molecules
25	LysoPE(0:0/20:3(11Z,14Z,17Z))	$C_{25}H_{46}NO_7P$	0.48	Lipids and lipid-like molecules

26	Bis(2-methylundecan-2-yl) Disulfide	$C_{24}H_{50}S_2$	1.34	Organosulfur compounds
27	N-Arachidonoyl-3-hydroxy-gamma-Aminobutyric Acid	$C_{24}H_{39}NO_4$	1.29	-
28	Alpha-Cadinene	$C_{15}H_{24}$	1.48	Lipids and lipid-like molecules
29	(-)-Variabilin	$C_{17}H_{16}O_5$	1.69	-
30	Propyl gallate	$C_{10}H_{12}O_5$	0.73	Benzenoids
31	Crispolide	$C_{15}H_{20}O_5$	0.37	Lipids and lipid-like molecules
32	Grevilline A	$C_{18}H_{12}O_6$	2.18	Organoheterocyclic compounds
33	CP 47,497-C8-homolog C-8-hydroxy metabolite	$C_{22}H_{36}O_3$	1.56	-
34	2-amino-14,16-dimethyloctad	$C_{20}H_{43}NO$	2.72	-

	ecan-3-ol			
35	Sphingosine	$C_{18}H_{37}NO_2$	1.56	Organic nitrogen compounds
36	Sorbitan oleate	$C_{24}H_{44}O_6$	0.08	Lipids and lipid-like molecules
37	Eupatorin	$C_{18}H_{16}O_7$	1.95	-
38	(3E,5Z)-1,3,5-Heptatriene	C_7H_{10}	1.89	Hydrocarbons
39	Methyl (R)-9-hydroxy-10-undecene-5,7-diynoate glucoside	$C_{18}H_{24}O_8$	1.39	Lipids and lipid-like molecules
40	(25R)-3beta-hydroxycholest-5-en-7-one-26-oate	$C_{27}H_{42}O_4$	1.44	Lipids and lipid-like molecules
41	(1S)-1-hydroxy-23-oxo-24,25,26,27-tetranorcalciol	$C_{23}H_{34}O_3$	1.93	-

42	N1-Acetylspermine	$C_{12}H_{28}N_4O$	4.33	Organic acids and derivatives
43	1-Hexadecanol	$C_{16}H_{34}O$	1.51	Lipids and lipid-like molecules
44	Lysyl-Lysine	$C_{12}H_{26}N_4O_3$	1.18	Organic acids and derivatives
45	Glycidyl oleate	$C_{21}H_{38}O_3$	3.15	-
46	5(S),6(S)-DiHETE	$C_{20}H_{32}O_4$	4.87	-
47	(24R)-Ergost-4-ene-3,6-dione	$C_{28}H_{44}O_2$	2.26	Lipids and lipid-like molecules
48	()12,13-DiHOME	$C_{18}H_{34}O_4$	2.14	-
49	Octadecanol	$C_{18}H_{38}O$	3.03	Lipids and lipid-like molecules
50	Avenalumic acid	$C_{11}H_{10}O_3$	1.98	Benzenoids
51	N2-Galacturonyl-L-lysine	$C_{12}H_{22}N_2O_8$	1.98	Organic acids and derivatives
52	Hypoxanthine	$C_5H_4N_4O$	70.57	Organoheterocyclic compounds

53	Pinostrobin	$C_{16}H_{14}O_4$	1.50	-
54	(all-E)-1,7,9-Heptadecatriene-11,13,15-triyne	$C_{17}H_{18}$	1.59	Hydrocarbons
55	Piperine	$C_{17}H_{19}NO_3$	1.51	Alkaloids and derivatives
56	Methoxyeugenol	$C_{11}H_{14}O_3$	1.35	Benzenoids
57	Avenanthramide A2	$C_{18}H_{19}NO_7$	1.59	Phenylpropanoids and polyketides
58	N-(14-Methylhexadecanoyl)pyrrolidine	$C_{21}H_{41}NO$	1.69	Organoheterocyclic compounds
59	1-(beta-D-Ribofuranosyl)-1,4-dihydronicotinamide	$C_{11}H_{16}N_2O_5$	0.70	Organic oxygen compounds
60	Trans-Grandmarin	$C_{15}H_{16}O_6$	0.74	Phenylpropanoids and polyketides
61	L-Urobilinogen	$C_{33}H_{48}N_4O_6$	1.45	Organoheterocyclic compounds

62	1-Oleoylglycerophosphoinositol	$C_{27}H_{51}O_{12}P$	0.48	Lipids and lipid-like molecules
63	Polyoxyethylene (600) mono-ricinoleate	$C_{21}H_{40}O_3$	2.33	-
64	LysoPE(0:0/20:2(11Z,14Z))	$C_{25}H_{48}NO_7P$	0.32	Lipids and lipid-like molecules
65	N,N-dimethyl-L-Valine	$C_7H_{15}NO_2$	1.18	-
66	Longifolenaldehyde	$C_{15}H_24O$	1.64	-
67	2-Methylbutylamine	$C_5H_{13}N$	1.55	Organic nitrogen compounds
68	3,4-Dihydro-4-[(5-methyl-2-furanyl)methylene]-2H-pyrrole	$C_{10}H_{11}NO$	1.24	Organoheterocyclic compounds
69	Tyrosyl-Hydroxyproline	$C_{14}H_{18}N_2O_5$	0.52	Organic acids and derivatives

70	N-oleoyl threonine	$C_{22}H_{41}NO_4$	1.31	-
71	Mangiferic acid	$C_{18}H_{32}O_2$	5.19	Lipids and lipid-like molecules
72	Isopentenyl adenosine	$C_{15}H_{21}N_5O_4$	1.24	-
73	2-Dehydro-O-desmethylangol ensin	$C_{15}H_{12}O_4$	1.85	Phenylpropanoids and polyketides
74	7-beta-D-Glucopyranosyloxy butylidenephthalide	$C_{18}H_{22}O_8$	1.25	Organic oxygen compounds
75	2-octenoylglycine	$C_{10}H_{17}NO_3$	1.39	Organic acids and derivatives
76	ALANYL-dl-PHENYLALA NINE	$C_{12}H_{16}N_2O_3$	0.44	-
77	(+-)-Ethyl 3-hydroxy-2-methylbutyrate	$C_{30}H_{48}O_3$	1.36	Lipids and lipid-like molecules

78	PC(O-6:0/6:0)[U]	$C_{20}H_{42}NO_7P$	0.56	-
79	Octadecylamine	$C_{18}H_{39}N$	1.20	Organic nitrogen compounds
80	S-(1,2-DICARBOXYETHYL)GLUTATHIONE	$C_{14}H_{21}N_3O_{10}S$	1.20	-
81	11Z-hexadecenoic acid	$C_{16}H_{30}O_2$	1.48	-
82	S-Adenosylhomocysteine	$C_{14}H_{20}N_6O_5S$	1.17	Nucleosides, nucleotides, and analogues
83	1alpha,3alpha,7alpha-Trihydr oxy-5beta-cholan-24-oic Acid	$C_{24}H_{40}O_5$	1.15	-
84	Vulgarolide	$C_{15}H_{20}O_5$	1.33	Lipids and lipid-like molecules
85	(+)-Galeon	$C_{20}H_{22}O_4$	1.52	Phenylpropanoids and polyketides
86	Dehydronuciferine	$C_{19}H_{19}NO_2$	1.78	Organic acids and derivatives

87	Acetylhomoserine	$C_6H_{11}NO_4$	1.22	Organic acids and derivatives
88	Oxymetholone	$C_{21}H_{32}O_3$	2.31	-
89	Serratol	$C_{20}H_{34}O$	1.39	Lipids and lipid-like molecules
90	LysoPA(0:0/18:2(9Z,12Z))	$C_{21}H_{39}O_7P$	0.46	Lipids and lipid-like molecules
91	Methyl (Z,Z)-10-hydroxy-2,8-decadiene-4,6-diynoate	$C_{11}H_{10}O_3$	1.35	Lipids and lipid-like molecules
92	12S-HHT	$C_{17}H_{28}O_3$	1.40	Lipids and lipid-like molecules
93	PC(15:1(9Z)/0:0)	$C_{23}H_{46}NO_7P$	0.53	-
94	9(S)-HOTrE	$C_{18}H_{30}O_3$	1.91	-
95	L-Pyridosine	$C_{12}H_{18}N_2O_4$	0.85	Organic acids and derivatives

96	Dihydroactinidiolide	$C_{11}H_{16}O_2$	1.21	Organoheterocyclic compounds
97	Genistein	$C_{15}H_{10}O_5$	3.53	Phenylpropanoids and polyketides
98	3(4->5)-Abeo-4,11:4,12-diepoxy-3-eudesmanol	$C_{15}H_{24}O_3$	1.29	Organoheterocyclic compounds
99	Budesonide	$C_{25}H_{34}O_6$	1.20	Lipids and lipid-like molecules
100	PS(18:1(9Z)/0:0)	$C_{24}H_{46}NO_9P$	0.31	-
101	Oleoyl Ethanolamide	$C_{20}H_{39}NO_2$	1.19	-
102	Vinblastine	$C_{46}H_{58}N_4O_9$	4.62	Organic acids and derivatives
103	Maysin 3'-methyl ether	$C_{28}H_{30}O_{14}$	0.72	Phenylpropanoids and polyketides
104	2-Methoxy-3-(4-methoxyphenyl)propanoic acid	$C_{11}H_{14}O_4$	1.48	Phenylpropanoids and polyketides

105	Tangeritin	$C_{20}H_{20}O_7$	1.23	Phenylpropanoids and polyketides
106	Nobiletin	$C_{21}H_{22}O_8$	1.16	Phenylpropanoids and polyketides
107	PG(16:1(9Z)/18:1(11Z))	$C_{40}H_{75}O_{10}P$	1.89	Lipids and lipid-like molecules
108	(9Me,4E,8E,10E-d19:3)sphingosine	$C_{19}H_{35}NO_2$	1.21	-
109	(4E)-12-hydroxy-1-(4-hydroxy-3-methoxyphenyl)dodec-4-en-3-one	$C_{19}H_{28}O_4$	1.26	Benzenoids
110	Eremopetasidione	$C_{14}H_{20}O_3$	1.36	Lipids and lipid-like molecules
111	Docosatrienoic acid	$C_{22}H_{38}O_2$	1.20	Lipids and lipid-like molecules
112	Alectrol	$C_{19}H_{22}O_6$	1.61	Organoheterocyclic compounds
113	HYMECROMONE	$C_{11}H_{10}O_3$	0.29	-

	METHYL ETHER			
114	19-Oxoandrost-4-ene-3,17-di one	$C_{19}H_{24}O_3$	1.26	Lipids and lipid-like molecules
115	N1,N8-Diacetylspermidine	$C_{11}H_{23}N_3O_2$	9.31	Organic acids and derivatives
116	Tyrosyl-Alanine	$C_{12}H_{16}N_2O_4$	1.24	Organic acids and derivatives
117	Inosine	$C_{10}H_{12}N_4O_5$	11.79	Nucleosides, nucleotides, and analogues
118	MG(16:1(9Z)/0:0/0:0)	$C_{19}H_{36}O_4$	0.65	Lipids and lipid-like molecules
119	1-Monopalmitin	$C_{19}H_{38}O_4$	0.52	-
120	ALANYL-dl-LEUCINE	$C_9H_{18}N_2O_3$	0.48	-
121	Polyporusterone F	$C_{28}H_{46}O_5$	1.23	Lipids and lipid-like molecules
122	3-(4-Methylphenyl)oxiraneca	$C_{10}H_{10}O_3$	1.32	Benzenoids

	rboxylic acid			
123	PE(18:1(9Z)/0:0)	$C_{23}H_{46}NO_7P$	0.64	-
124	Spermine	$C_{10}H_{26}N_4$	3.92	Organic nitrogen compounds
125	Farnesyl acetone	$C_{18}H_{30}O$	0.60	-
126	9S-hydroxy-12R,13S-epoxy-10E,15Z-octadecadienoic acid	$C_{18}H_{30}O_4$	1.18	-
127	Alpha-9(10)-EpODE	$C_{18}H_{30}O_3$	1.15	-
128	Atalantoflavone	$C_{20}H_{16}O_5$	1.38	Phenylpropanoids and polyketides
129	2-Pyrrolidone-5-carboxylic acid, methyl ester	$C_6H_9NO_3$	2.14	-
130	Palmitic amide	$C_{16}H_{33}NO$	1.22	Lipids and lipid-like molecules

131	Gamma-Tocotrienol	$C_{28}H_{42}O_2$	1.24	Lipids and lipid-like molecules
132	Adenine	$C_5H_5N_5$	0.80	Organoheterocyclic compounds
133	Sphinganine	$C_{18}H_{39}NO_2$	1.17	Organic nitrogen compounds
134	6a-Hydroxyestrone	$C_{18}H_{22}O_3$	1.48	-
135	DIHYDRO TANSHINONE	$C_{18}H_{14}O_3$	1.17	-
136	Angiotensin IV	$C_{40}H_{54}N_8O_8$	2.09	Nucleosides, nucleotides, and analogues
137	MG(18:1(9Z)/0:0/0:0)[rac]	$C_{21}H_{40}O_4$	0.72	-
138	1-Linoleoyl Glycerol	$C_{21}H_{38}O_4$	0.73	-
139	N-lactoyl-phenylalanine	$C_{12}H_{15}NO_4$	1.13	Organic acids and derivatives
140	L-Carnitine	$C_7H_{15}NO_3$	1.14	Organic nitrogen compounds
141	Spisulosine	$C_{18}H_{39}NO$	1.18	-

142	28:7(n-6)	$C_{28}H_{42}O_2$	1.20	-
143	LysoPE(16:1(9Z)/0:0)	$C_{21}H_{42}NO_7P$	0.55	Lipids and lipid-like molecules
144	Ricinoleic acid	$C_{18}H_{34}O_3$	1.93	Lipids and lipid-like molecules
145	Phytosphingosine	$C_{18}H_{39}NO_3$	1.15	Organic nitrogen compounds
146	(6alpha,22E)-6-Hydroxy-4,7,22-ergostatrien-3-one	$C_{28}H_{42}O_2$	1.28	Lipids and lipid-like molecules
147	PE(16:0/0:0)	$C_{21}H_{44}NO_7P$	0.63	-
148	PA(16:1(9Z)/0:0)	$C_{19}H_{37}O_7P$	0.30	-
149	1-Oleoyl Lysophosphatidic Acid (sodium salt)	$C_{21}H_{41}O_7P$	0.75	-
150	6-hydroxy-7E,9E-Octadecadiene-11,13,15,17-tetraynoic	$C_{18}H_{16}O_3$	1.16	-

	acid			
151	2,2,4-Trimethyl-6-(1-oxo-3-phenylpropyl)-1,3,5-cyclohexanetrione	$C_{18}H_{20}O_4$	1.12	-
152	()9-HpODE	$C_{18}H_{32}O_4$	1.19	-
153	13-Hydroxyhexadecanoic acid	$C_{16}H_{32}O_3$	1.11	Lipids and lipid-like molecules
154	Spermidine	$C_7H_{19}N_3$	1.21	Organic nitrogen compounds
155	N,N-dimethyl-Safingol	$C_{20}H_{43}NO_2$	1.09	-
156	LysoPC(18:1(9Z))	$C_{26}H_{52}NO_7P$	0.72	Lipids and lipid-like molecules
157	1-Palmitoyl Lysophosphatidic Acid	$C_{19}H_{39}O_7P$	0.65	-

158	Oleamide	$C_{18}H_{35}NO$	1.18	Lipids and lipid-like molecules
159	PC(16:1(9Z)/18:1(11Z))	$C_{42}H_{80}NO_8P$	1.17	Lipids and lipid-like molecules
160	PC(16:1(9Z)/16:1(9Z))	$C_{40}H_{76}NO_8P$	1.13	Lipids and lipid-like molecules
161	PC(16:0/0:0)[U]	$C_{24}H_{50}NO_7P$	0.65	-
162	1-Linoleoylglycerophosphocholine	$C_{26}H_{50}NO_7P$	0.59	Lipids and lipid-like molecules
163	LPA(18:2(9Z,12Z)/0:0)	$C_{21}H_{39}O_7P$	0.64	-
164	2-Linoleoyl Glycerol	$C_{21}H_{38}O_4$	0.75	-
165	1-Palmitoyl-2-linoleoyl PE	$C_{39}H_{74}NO_8P$	1.26	-
166	PE(16:1(5Z)/16:1(5Z))	$C_{37}H_{70}NO_8P$	1.21	-
167	13Z-Docosenamide	$C_{22}H_{43}NO$	1.18	-

168	MG(0:0/18:0/0:0)	$C_{21}H_{42}O_4$	1.15	Lipids and lipid-like molecules
169	9,10-DiHOME	$C_{18}H_{34}O_4$	1.31	-
170	DL-2-hydroxy stearic acid	$C_{18}H_{36}O_3$	1.28	-
171	2-Carboxy-4-dodecanolide	$C_{13}H_{22}O_4$	1.32	Organoheterocyclic compounds
172	9(S)-HpODE	$C_{18}H_{32}O_4$	1.17	Lipids and lipid-like molecules
173	Hexadecanedioic acid	$C_{16}H_{30}O_4$	1.37	Lipids and lipid-like molecules
174	Dolichosterone	$C_{28}H_{46}O_5$	1.41	Lipids and lipid-like molecules
175	All-trans-heptaprenyl diphosphate	$C_{35}H_{60}O_7P_2$	0.49	Lipids and lipid-like molecules
176	Cortol	$C_{21}H_{36}O_5$	0.58	Lipids and lipid-like molecules
177	Lactosylceramide	$C_{42}H_{79}NO_{13}$	1.30	Lipids and lipid-like molecules

	(d18:1/12:0)			
178	Fasciculic acid A	$C_{36}H_{60}O_8$	1.89	Lipids and lipid-like molecules
179	PE(15:0/22:1(13Z))	$C_{42}H_{82}NO_8P$	1.36	Lipids and lipid-like molecules
180	PE(15:0/22:2(13Z,16Z))	$C_{42}H_{80}NO_8P$	1.16	Lipids and lipid-like molecules
181	PE(15:0/20:2(11Z,14Z))	$C_{40}H_{76}NO_8P$	1.13	Lipids and lipid-like molecules
182	16-Hydroxy hexadecanoic acid	$C_{16}H_{32}O_3$	1.30	Lipids and lipid-like molecules
183	13-Hydroxy-9-methoxy-10-oxo-11-octadecenoic acid	$C_{19}H_{34}O_5$	1.40	Lipids and lipid-like molecules
184	5'-Carboxy-gamma-chromanol	$C_{18}H_{26}O_4$	0.19	Organoheterocyclic compounds
185	L-(-)-3-Phenyllactic acid	$C_9H_{10}O_3$	1.46	-

186	Valyl-Aspartate	$C_9H_{16}N_2O_5$	0.80	Organic acids and derivatives
187	Valyl-Serine	$C_8H_{16}N_2O_4$	0.83	Organic acids and derivatives
188	Sonchuionoside C	$C_{19}H_{30}O_8$	0.87	Lipids and lipid-like molecules
189	N6-Acetyl-L-lysine	$C_8H_{16}N_2O_3$	0.72	Organic acids and derivatives
190	Serylisoleucine	$C_9H_{18}N_2O_4$	0.46	Organic acids and derivatives
191	(2-Methoxyethoxy)propanoic acid	$C_6H_{12}O_4$	0.81	Organic acids and derivatives
192	(S)-a-Amino-2,5-dihydro-5-oxo-4-isoxazolepropanoic acid N2-glucoside	$C_{12}H_{18}N_2O_9$	1.59	Organic oxygen compounds
193	Gamma-Glutamylglutamic acid	$C_{10}H_{16}N_2O_7$	0.88	Organic acids and derivatives

194	L-Tyrosine	$C_9H_{11}NO_3$	1.17	Organic acids and derivatives
195	Desmosine	$C_{24}H_{40}N_5O_8+$	2.73	Organic acids and derivatives
196	Arginyl-Phenylalanine	$C_{15}H_{23}N_5O_3$	0.75	Organic acids and derivatives
197	2-Hydroxybutyric acid	$C_4H_8O_3$	1.77	Organic acids and derivatives
198	Gamma-Glutamyltyrosine	$C_{14}H_{18}N_2O_6$	0.70	Organic acids and derivatives
199	DL-o-Tyrosine	$C_9H_{11}NO_3$	1.74	-
200	Valyl-Methionine	$C_{10}H_{20}N_2O_3S$	0.84	Organic acids and derivatives
201	Glycyl-Isoleucine	$C_8H_{16}N_2O_3$	0.63	Organic acids and derivatives
202	Methionyl-Tyrosine	$C_{14}H_{20}N_2O_4S$	0.54	Organic acids and derivatives
203	Glutamylvaline	$C_{10}H_{18}N_2O_5$	0.74	Organic acids and derivatives
204	Isoleucyl-Tryptophan	$C_{17}H_{23}N_3O_3$	0.80	Organic acids and derivatives

205	Acetyl-DL-Leucine	$C_8H_{15}NO_3$	1.56	-
206	Indolelactic acid	$C_{11}H_{11}NO_3$	5.47	Organoheterocyclic compounds
207	S-Formylglutathione	$C_{11}H_{17}N_3O_7S$	1.30	Organic acids and derivatives
208	Cyclocalopin F	$C_{15}H_{18}O_6$	1.44	Organoheterocyclic compounds
209	Polyethylene, oxidized	$C_{12}H_{20}O_5$	1.16	Organic acids and derivatives
210	5-Hydroxy-L-tryptophan	$C_{11}H_{12}N_2O_3$	1.57	Organoheterocyclic compounds
211	Monic acid	$C_{18}H_{30}O_6$	0.73	Lipids and lipid-like molecules
212	5-Hydroxy-3',4',7,8-tetrameth oxyflavone	$C_{19}H_{18}O_7$	1.56	Phenylpropanoids and polyketides
213	Glycylproline	$C_7H_{12}N_2O3$	0.79	Organic acids and derivatives
214	2-Hydroxy-2,6,6-trimethylcyc	$C_9H_{16}O_2$	1.19	Organic oxygen compounds

	lohexanone			
215	Diosmetin	$C_{16}H_{12}O_6$	2.43	Phenylpropanoids and polyketides
216	(2S,2'S)-Pyrosaccharopine	$C_{11}H_{18}N_2O_5$	1.58	Organic acids and derivatives
217	Ochratoxin B	$C_{20}H_{19}NO_6$	1.62	Phenylpropanoids and polyketides
218	2,2'-(3-methylcyclohexane-1, 1-diyl)diacetic acid	$C_{11}H_{18}O_4$	1.36	-
219	Hydroxytanshinone	$C_{19}H_{18}O_4$	4.07	Lipids and lipid-like molecules
220	Isoliquiritigenin	$C_{15}H_{12}O_4$	3.44	Phenylpropanoids and polyketides
221	Traumatic acid	$C_{12}H_{20}O_4$	1.36	Lipids and lipid-like molecules
222	Carnosic acid	$C_{20}H_{28}O_4$	1.30	Lipids and lipid-like molecules
223	Neocnidilide	$C_{12}H_{18}O_2$	3.90	Organoheterocyclic compounds

224	Tryptophyl-Hydroxyproline	$C_{16}H_{19}N_3O_4$	0.78	Organic acids and derivatives
225	(17alpha,23S)-17,23-Epoxy-29-hydroxy-27-norlanosta-1,8-diene-3,15,24-trione	$C_{29}H_{40}O_5$	0.82	Lipids and lipid-like molecules
226	6-Keto-prostaglandin F1a	$C_{20}H_{34}O_6$	1.25	Lipids and lipid-like molecules
227	7-Hydroxy-5-(4-hydroxy-2-oxopentyl)-2-methylchromone 7-glucoside	$C_{21}H_{26}O_{10}$	1.25	Organic oxygen compounds
228	Heptaethylene glycol	$C_{14}H_{30}O_8$	1.24	Organic oxygen compounds
229	2'-Hydroxyenterolactone	$C_{18}H_{18}O_5$	1.19	Lignans, neolignans and related compounds
230	N-(1-Deoxy-1-fructosyl)tyrosine	$C_{15}H_{21}NO_8$	1.40	Organic acids and derivatives

231	Mangalkanyl glucoside	$C_{21}H_{38}O_6$	0.08	Organic oxygen compounds
232	Melleolide D	$C_{24}H_{31}ClO_8$	1.32	Lipids and lipid-like molecules
233	MG(0:0/20:4(8Z,11Z,14Z,17Z)/0:0)	$C_{23}H_{38}O_4$	1.37	Lipids and lipid-like molecules
234	9-Pentadecenoic acid	$C_{15}H_{28}O_2$	1.54	Lipids and lipid-like molecules
235	5,8-Dimethoxychalepensin	$C_{18}H_{18}O_5$	2.00	Phenylpropanoids and polyketides
236	Honokiol	$C_{18}H_{18}O_2$	1.38	-
237	Bassic acid	$C_{30}H_{46}O_5$	1.29	Lipids and lipid-like molecules
238	5,7-dihydroxy-3,6,8-trimethoxy-2-phenyl-4H-chromen-4-one	$C_{18}H_{16}O_7$	1.47	Phenylpropanoids and polyketides
239	15-Deoxy-d-12,14-PGJ2	$C_{20}H_{28}O_3$	1.63	Lipids and lipid-like molecules

240	Gymnodimine	$C_{32}H_{45}NO_4$	2.47	Organoheterocyclic compounds
241	Fumonisin B4	$C_{34}H_{59}NO_{13}$	0.63	Organic acids and derivatives
242	3'-Amino-3'-deoxythimidine glucuronide	$C_{16}H_{23}N_3O_{10}$	1.32	Nucleosides, nucleotides, and analogues
243	5-Tetradecenoic acid	$C_{14}H_{26}O_2$	1.54	Lipids and lipid-like molecules
244	Digoxigenin monodigitoxoside	$C_{29}H_{44}O_8$	1.30	Organic oxygen compounds
245	3b-Hydroxy-6b-angeloyloxy-7(11)-eremophilen-12,8b-olide	$C_{20}H_{28}O_5$	1.51	Lipids and lipid-like molecules
246	LysoPC(18:2(9Z,12Z))	$C_{26}H_{50}NO_7P$	0.16	Lipids and lipid-like molecules
247	24,25,26,27-Tetranor-23-oxo-	$C_{23}H_{34}O_3$	1.44	Lipids and lipid-like molecules

	hydroxyvitamin D3			
248	[12]-Gingerol	$C_{23}H_{38}O_4$	5.69	Benzenoids
249	Gingerglycolipid B	$C_{33}H_{58}O_{14}$	0.69	Lipids and lipid-like molecules
250	Cyclopassifloic acid B	$C_{31}H_{52}O_6$	0.57	Lipids and lipid-like molecules
251	15(S)-Hydroxyeicosatrienoic acid	$C_{20}H_{34}O_3$	0.24	Lipids and lipid-like molecules
252	Asteltoxin	$C_{23}H_{30}O_7$	1.64	Organoheterocyclic compounds
253	8-Oxohexadecanoic acid	$C_{16}H_{30}O_3$	1.26	Lipids and lipid-like molecules
254	Gingerglycolipid C	$C_{33}H_{60}O_{14}$	0.79	Lipids and lipid-like molecules
255	Oleic acid	C18H34O2	2.51	Lipids and lipid-like molecules
256	Norfloxacin	$C_{16}H_{18}FN_3O_3$	0.11	Organoheterocyclic compounds

257	1-docosanoyl-glycero-3-phosphate	$C_{25}H_{51}O_7P$	0.18	Lipids and lipid-like molecules
258	1-Stearoylglycerophosphoserine	$C_{24}H_{48}NO_9P$	0.25	Lipids and lipid-like molecules
259	Abietinal	$C_{20}H_{30}O$	1.37	Lipids and lipid-like molecules
260	1-Acetoxy-2-hydroxy-5,12,15-heneicosatrien-4-one	$C_{23}H_{38}O_4$	0.35	Lipids and lipid-like molecules
261	Cytochalasin Opho	$C_{28}H_{37}NO_4$	0.11	Alkaloids and derivatives
262	PE(18:1(11Z)/22:2(13Z,16Z))	$C_{45}H_{84}NO_8P$	1.31	Lipids and lipid-like molecules
263	27-Hydroxybullatacin	$C_{37}H_{66}O_8$	3.19	Lipids and lipid-like molecules
264	PE(15:0/14:0)	$C_{34}H_{68}NO_8P$	2.05	Lipids and lipid-like molecules
265	Stearoyllactic acid	$C_{21}H_{40}O_4$	1.94	Lipids and lipid-like molecules

266	PE(15:0/18:2(9Z,12Z))	$C_{38}H_{72}NO_8P$	1.30	Lipids and lipid-like molecules
267	Stearic Acid ethyl ester	$C_{20}H_{40}O_2$	3.33	-
268	PE(16:0/18:1(11Z))	$C_{39}H_{76}NO_8P$	1.38	Lipids and lipid-like molecules
269	PE(16:1(9Z)/20:1(11Z))	$C_{41}H_{78}NO_8P$	3.78	Lipids and lipid-like molecules
270	PS(15:0/18:1(11Z))	$C_{39}H_{74}NO_{10}P$	1.75	Lipids and lipid-like molecules
271	PE(15:0/20:1(11Z))	$C_{40}H_{78}NO_8P$	1.19	Lipids and lipid-like molecules
272	PC(14:1(9Z)/22:2(13Z,16Z))	$C_{44}H_{82}NO_8P$	2.55	Lipids and lipid-like molecules
273	PA(18:1(11Z)/16:1(9Z))	$C_{37}H_{69}O_8P$	1.28	Lipids and lipid-like molecules
274	PS(18:0/18:1(9Z))	$C_{42}H_{80}NO_{10}P$	2.22	Lipids and lipid-like molecules
275	PE(16:0/20:1(11Z))	$C_{41}H_{80}NO_8P$	1.64	Lipids and lipid-like molecules
276	PS(16:0/16:0)	$C_{38}H_{74}NO_{10}P$	2.06	Lipids and lipid-like molecules

277	PS(DiMe(11,3)/DiMe(13,5))	$C_{50}H_{86}NO_{12}P$	1.43	Lipids and lipid-like molecules
278	PE(18:1(11Z)/18:1(11Z))	$C_{41}H_{78}NO_8P$	1.27	Lipids and lipid-like molecules
279	PE-NMe2(14:0/16:1(9Z))	$C_{37}H_{72}NO_8P$	2.20	Lipids and lipid-like molecules
280	PE-NMe(15:0/15:0)	$C_{36}H_{72}NO_8P$	1.51	Lipids and lipid-like molecules
281	PE(16:1(9Z)/18:1(11Z))	$C_{39}H_{74}NO_8P$	1.26	Lipids and lipid-like molecules
282	Ginsenoside F2	$C_{42}H_{72}O_{13}$	1.69	Lipids and lipid-like molecules
283	Physapubescin	$C_{30}H_{42}O_8$	0.59	Lipids and lipid-like molecules
284	PE(15:0/16:1(9Z))	$C_{36}H_{70}NO_8P$	1.26	Lipids and lipid-like molecules
285	Ascorbyl stearate	$C_{24}H_{42}O_7$	1.42	Lipids and lipid-like molecules
286	PE(14:1(9Z)/15:0)	$C_{34}H_{66}NO_8P$	1.51	Lipids and lipid-like molecules
287	Octadecanedioic acid	$C_{18}H_{34}O_4$	0.76	Lipids and lipid-like molecules

288	Deoxycholic acid glycine conjugate	$C_{26}H_{43}NO_5$	1.57	Lipids and lipid-like molecules
289	Sagittariol	$C_{20}H_{34}O_2$	1.52	Lipids and lipid-like molecules
290	(+)-15,16-Dihydroxyoctadecanoic acid	$C_{18}H_{36}O_4$	0.48	Lipids and lipid-like molecules
291	(E)-3-Hexadecenoic acid	$C_{16}H_{30}O_2$	1.23	Lipids and lipid-like molecules
292	LysoPC(18:0)	$C_{26}H_{54}NO_7P$	0.84	Lipids and lipid-like molecules
293	2-hydroxyhexadecanoic acid	$C_{16}H_{32}O_3$	1.13	Lipids and lipid-like molecules
294	1-(9Z,12Z,15Z-octadecatrienoyl)-glycero-3-phosphate	$C_{21}H_{37}O_7P$	0.16	Lipids and lipid-like molecules
295	Margaroylglycine	$C_{19}H_{37}NO_3$	1.38	Organic acids and derivatives
296	Sarcodon scabrosus	$C_{23}H_{39}N_3O_8$	1.42	Organic acids and derivatives

	Depsipeptide			
297	4Alpha-hydroxymethyl-5alpha-cholesta-8,24-dien-3beta-ol	$C_{28}H_{46}O_2$	1.53	Lipids and lipid-like molecules
298	Palmitoyl glucuronide	$C_{22}H_{42}O_7$	0.28	Lipids and lipid-like molecules
299	Dihydroceramide	$C_{19}H_{39}NO_3$	1.48	Organic acids and derivatives
300	Quillaic acid 3-[rhamnosyl-(1->3)-[galactosyl-(1->2)]-glucuronide]	$C_{48}H_{74}O_{20}$	3.68	Lipids and lipid-like molecules
301	MG(0:0/18:3(9Z,12Z,15Z)/0:0)	$C_{21}H_{36}O_4$	0.22	Lipids and lipid-like molecules
302	Vitamin D2 3-glucuronide	$C_{34}H_{52}O_7$	0.89	Lipids and lipid-like molecules
303	Trigoneoside XIb	$C_{44}H_{74}O_{19}$	0.77	Lipids and lipid-like molecules

304	Janthitrem F	$C_{39}H_{51}NO_7$	0.09	Organoheterocyclic compounds
305	Hexadecyl ferulate	$C_{26}H_{42}O_4$	1.42	Phenylpropanoids and polyketides
306	25-Hydroxyvitamin D3-26,23-lactol	$C_{27}H_{42}O_4$	1.84	Lipids and lipid-like molecules
307	LysoPC(16:0)	$C_{24}H_{50}NO_7P$	0.60	Lipids and lipid-like molecules
308	Cyclohexaneundecanoic acid	$C_{17}H_{32}O_2$	1.43	Lipids and lipid-like molecules
309	1-Oleoylglycerophosphoserine	$C_{24}H_{46}NO_9P$	0.05	Lipids and lipid-like molecules
310	Fluticasone 17beta-carboxylic acid	$C_{21}H_{26}F_2O_5$	1.31	Lipids and lipid-like molecules
311	Armillaric acid	$C_{23}H_{28}O_7$	2.66	Lipids and lipid-like molecules
312	LysoPE(0:0/20:1(11Z))	$C_{25}H_{50}NO_7P$	0.61	Lipids and lipid-like molecules

313	LysoPE(18:2(9Z,12Z)/0:0)	$C_{23}H_{44}NO_7P$	0.76	Lipids and lipid-like molecules
314	(-)-Usnic acid	$C_{18}H_{16}O_7$	1.62	-
315	Dolicholide	$C_{28}H_{46}O_6$	1.37	Organic oxygen compounds
316	1-Formylneogrifolin	$C_{23}H_{32}O_3$	2.60	Lipids and lipid-like molecules
317	7a,12a-Dihydroxy-3-oxo-4-cholenoic acid	$C_{24}H_{36}O_5$	1.39	Lipids and lipid-like molecules
318	Gingerglycolipid A	$C_{33}H_{56}O_{14}$	0.14	Lipids and lipid-like molecules
319	3-Hydroxydodecanoic acid	$C_{12}H_{24}O_3$	1.44	Organic acids and derivatives
320	Betavulgaroside VI	$C_{47}H_{72}O_{21}$	0.60	Lipids and lipid-like molecules
321	13,14-Dihydro PGE1	$C_{20}H_{36}O_5$	15.42	Lipids and lipid-like molecules
322	Calcitroic acid	$C_{23}H_{34}O_4$	1.34	Lipids and lipid-like molecules

323	Auberganol	$C_{15}H_{28}O_2$	0.62	Lipids and lipid-like molecules
324	Prostaglandin F3a	$C_{20}H_{32}O_5$	1.68	Lipids and lipid-like molecules
325	Emodin	$C_{15}H_{10}O_5$	1.72	Benzenoids
326	(6b,7b,13R)-6,7-Diacetoxy-8,14-labdadiene-13-ol	$C_{24}H_{38}O_5$	1.39	Lipids and lipid-like molecules
327	MG(0:0/20:5(5Z,8Z,11Z,14Z,17Z)/0:0)	$C_{23}H_{36}O_4$	1.27	Lipids and lipid-like molecules
328	2-Dodecylbenzenesulfonic acid	$C_{18}H_{30}O_3S$	3.14	Benzenoids
329	LysoPE(15:0/0:0)	$C_{20}H_{42}NO_7P$	0.67	Lipids and lipid-like molecules
330	METHYLNORLICHEXANTHONE	$C_{15}H_{12}O_5$	1.86	-

331	10-Undecen-2-one	$C_{11}H_{20}O$	8.00	Organic oxygen compounds
332	12-Oxo-20-hydroxy-leukotriene B4	$C_{20}H_{30}O_5$	1.78	Lipids and lipid-like molecules
333	Cloversaponin I	$C_{36}H_{56}O_9$	1.19	Lipids and lipid-like molecules
334	Tetradecanedioic acid	$C_{14}H_{26}O_4$	1.45	Lipids and lipid-like molecules
335	Achillicin	$C_{17}H_{22}O_5$	1.78	Organoheterocyclic compounds
336	Floionolic acid	$C_{18}H_{36}O_5$	2.57	Lipids and lipid-like molecules
337	12-Oxo-20-carboxy-leukotriene B4	$C_{20}H_{28}O_6$	1.38	Lipids and lipid-like molecules
338	(-)-Nopol	$C_{11}H_{18}O$	2.40	Lipids and lipid-like molecules
339	2-Decarboxybetanin	$C_{23}H_{27}N_2O_{11}+$	1.48	Organic oxygen compounds

340	9,10,13-TriHOME	$C_{18}H_{34}O_5$	1.24	Lipids and lipid-like molecules
341	5,8,12-Trihydroxy-9-octadecenoic acid	$C_{18}H_{34}O_5$	2.20	Lipids and lipid-like molecules
342	Garcinone B	$C_{23}H_{22}O_6$	1.24	Organoheterocyclic compounds
343	Perulactone B	$C_{28}H_{40}O_7$	0.76	Lipids and lipid-like molecules
344	2-Phenylaminoadenosine	$C_{16}H_{18}N_6O_4$	1.38	Nucleosides, nucleotides, and analogues
345	3-Methylbutyl 2-furanbutanoate	$C_{13}H_{20}O_3$	1.39	Lipids and lipid-like molecules
346	Armillaridin	$C_{24}H_{29}ClO_6$	1.26	Lipids and lipid-like molecules
347	Cryptomeridiol 11-rhamnoside	$C_{21}H_{38}O_6$	0.19	Lipids and lipid-like molecules
348	2-hydroxy-3-[4-hydroxy-3-(3	$C_{14}H_{18}O_4$	1.34	Phenylpropanoids and polyketides

	-methylbut-2-en-1-yl)phenyl] propanoic acid			
349	Osmundalin	$C_{12}H_{18}O_8$	3.36	Organic oxygen compounds
350	11-nitro-1-undecene	$C_{11}H_{21}NO_2$	9.35	Organic 1,3-dipolar compounds
351	4-Hydroxy-2,6-dimethyl-7-octen-3-one	$C_{10}H_{18}O_2$	1.22	Organic oxygen compounds
352	4'-Hydroxy-R-phenprocoumon	$C_{18}H_{16}O_4$	1.53	Phenylpropanoids and polyketides
353	S-(Hydroxymethyl)glutathione	$C_{11}H_{19}N_3O_7S$	3.41	Organic acids and derivatives
354	2,3,6,7-Tetrahydro-7-methylcyclopent[b]azepin-8(1H)-one	$C_{10}H_{13}NO$	1.49	Organoheterocyclic compounds

355	Glyzarin	$C_{18}H_{14}O_4$	1.37	Phenylpropanoids and polyketides
356	Oryzalide B	$C_{19}H_{28}O_4$	0.05	Lipids and lipid-like molecules
357	(3'x,5'a,9'x,10'b)-O-(3-Hydroxy-6-oxo-7-drimen-11-yl)umbelliferone	$C_{24}H_{28}O_5$	0.55	Phenylpropanoids and polyketides
358	1-hydroxy-4-(4-hydroxy-3-methoxyphenyl)butan-2-one	$C_{11}H_{14}O_4$	2.16	Benzenoids
359	2,5-Dimethyl-4-ethoxy-3(2H)-furanone	$C_8H_{12}O_3$	1.49	Organoheterocyclic compounds
360	11-Dehydrocorticosterone	$C_{21}H_{28}O_4$	0.58	Lipids and lipid-like molecules
361	()-3-Hydroxynonanoic acid	$C_9H_{18}O_3$	1.36	Organic acids and derivatives
362	Gibberellin A38 glucosyl	$C_{26}H_{36}O_{11}$	0.86	Lipids and lipid-like molecules

	ester			
363	8-Acetylegelolide	$C_{16}H_{20}O_6$	1.19	Organoheterocyclic compounds
364	4-ene-Valproic acid	$C_8H_{14}O_2$	1.15	Lipids and lipid-like molecules
365	9'-Carboxy-gamma-tocotrienol	$C_{23}H_{32}O_4$	1.20	Lipids and lipid-like molecules
366	N-Acetyl-D-phenylalanine	$C_{11}H_{13}NO_3$	1.36	-
367	Glutamyltryptophan	$C_{16}H_{19}N_3O_5$	0.86	Organic acids and derivatives
368	Gamma-Glutamylphenylalanine	$C_{14}H_{18}N_2O_5$	0.35	Organic acids and derivatives
369	Glutamylisoleucine	$C_{11}H_{20}N_2O_5$	0.50	Organic acids and derivatives
370	L-1,2,3,4-Tetrahydro-beta-carboline-3-carboxylic acid	$C_{12}H_{12}N_2O_2$	1.50	Organoheterocyclic compounds

371	N-lactoyl-Methionine	$C_8H_{15}NO_4S$	2.26	Organic acids and derivatives
372	Hydroxyphenyllactic acid	$C_9H_{10}O_4$	1.22	Phenylpropanoids and polyketides
373	Threoninyl-Phenylalanine	$C_{13}H_{18}N_2O_4$	0.77	Organic acids and derivatives
374	Glycyl-Phenylalanine	C11H14N2O3	0.74	Organic acids and derivatives
375	Serylphenylalanine	$C1_2H_{16}N_2O_4$	0.00	Organic acids and derivatives
376	Biotripyrrin-a	$C_{25}H_{27}N_3O_6$	1.34	Organoheterocyclic compounds
377	Valyl-Tyrosine	$C_{14}H_{20}N_2O_4$	0.10	Organic acids and derivatives
378	Glutaminylvaline	$C_{10}H_{19}N_3O_4$	3.08	Organic acids and derivatives
379	Carbofuran	$C_{12}H_{15}NO_3$	2.63	Organoheterocyclic compounds
380	Oxoadipic acid	$C_6H_8O_5$	2.69	Organic acids and derivatives
381	Aspartyl-Tyrosine	$C_{13}H_{16}N_2O_6$	3.68	Organic acids and derivatives

382	Kanzonol F	$C_{26}H_{28}O_5$	1.43	Phenylpropanoids and polyketides
383	O-Desmethyl-lacosamide	$C_{12}H1_{6}N_2O_3$	0.77	Organic acids and derivatives
384	Pterolactam	$C_5H_9NO_2$	1.31	Organoheterocyclic compounds
385	Ophthalmic acid	$C1_{1}H_{19}N_3O_6$	0.19	Organic acids and derivatives
386	Asparaginyl-Phenylalanine	$C_{13}H_{17}N_3O_4$	0.11	Organic acids and derivatives
387	N-(1-Deoxy-1-fructosyl)glycine	$C_8H_{15}NO_7$	1.34	Organic oxygen compounds
388	Threoninyl-Isoleucine	$C_{10}H_{20}N_2O_4$	0.40	Organic acids and derivatives
389	Alanyl-Valine	$C_8H_{16}N_2O_3$	0.76	Organic acids and derivatives
390	3-Methyluridine	$C_{10}H_{14}N_2O_6$	1.95	Nucleosides, nucleotides, and analogues
391	Lysyl-Hydroxyproline	$C_{11}H_{21}N_3O_4$	0.60	Organic acids and derivatives

392	Uridine diphosphate (UDP)	$C_9H_{14}N_2O_{12}P_2$	1.33	-
393	8-[(Aminomethyl)sulfanyl]-6-sulfanyloctanoic acid	$C_9H_{19}NO_2S_2$	1.40	Lipids and lipid-like molecules
394	Gamma-Glu-Leu	$C_{11}H_{20}N_2O_5$	0.19	-
395	Isoleucyl-Glutamine	$C_{11}H_{21}N_3O_4$	0.56	Organic acids and derivatives
396	Valyl-Asparagine	$C_9H_{17}N_3O_4$	0.41	Organic acids and derivatives
397	2-Amino-4-[(2-hydroxy-1-oxopropyl)amino]butanoic acid	$C_7H_{14}N_2O_4$	0.01	Organic acids and derivatives
398	Pyrazin-2-carboxylic acid	$C_5H_4N_2O_2$	1.27	Organoheterocyclic compounds

Supplemental Table 3.

The information of total 104 DAMs.

	Metabolite	FC(F/UF)	HMDB Superclass
1	Hypoxanthine	70.57	Organoheterocyclic compounds
2	13,14-Dihydro PGE1	15.42	Lipids and lipid-like molecules
3	Inosine	11.79	Nucleosides, nucleotides, and analogues
4	11-nitro-1-undecene	9.35	Organic 1,3-dipolar compounds
5	N1,N8-Diacetylspermidine	9.31	Organic acids and derivatives
6	10-Undecen-2-one	8.00	Organic oxygen compounds
7	[12]-Gingerol	5.69	Benzenoids

8	Indolelactic acid	5.47	Organoheterocyclic compounds
9	Mangiferic acid	5.19	Lipids and lipid-like molecules
10	5(S),6(S)-DiHETE	4.87	-
11	Vinblastine	4.62	Organic acids and derivatives
12	N1-Acetylspermine	4.33	Organic acids and derivatives
13	Hydroxytanshinone	4.07	Lipids and lipid-like molecules
14	Spermine	3.92	Organic nitrogen compounds
15	Neocnidilide	3.90	Organoheterocyclic compounds
16	PE(16:1(9Z)/20:1(11Z))	3.78	Lipids and lipid-like molecules
17	Quillaic acid 3-[rhamnosyl-(1->3)-[galactosyl-(1->2)]-glucuro	3.68	Lipids and lipid-like molecules

	nide]		
18	Aspartyl-Tyrosine	3.68	Organic acids and derivatives
19	Doxercalciferol	3.58	-
20	Genistein	3.53	Phenylpropanoids and polyketides
21	Isoliquiritigenin	3.44	Phenylpropanoids and polyketides
22	S-(Hydroxymethyl)glutathione	3.41	Organic acids and derivatives
23	Osmundalin	3.36	Organic oxygen compounds
24	Stearic Acid ethyl ester	3.33	-
25	27-Hydroxybullatacin	3.19	Lipids and lipid-like molecules
26	Glycidyl oleate	3.15	-
27	2-Dodecylbenzenesulfonic acid	3.14	Benzenoids

28	Glutaminylvaline	3.08	Organic acids and derivatives
29	Octadecanol	3.03	Lipids and lipid-like molecules
30	PS(18:0/20:3(8Z,11Z,14Z))	2.88	Lipids and lipid-like molecules
31	Dihydrodeoxy-8-epiaustdiol	2.82	Organoheterocyclic compounds
32	Isopentyl gentiobioside	2.82	Lipids and lipid-like molecules
33	PA(16:0/18:2(9Z,12Z))	2.75	Lipids and lipid-like molecules
34	Desmosine	2.73	Organic acids and derivatives
35	2-amino-14,16-dimethyloctadecan-3-ol	2.72	-
36	Oxoadipic acid	2.69	Organic acids and derivatives
37	Armillaric acid	2.66	Lipids and lipid-like molecules
38	Carbofuran	2.63	Organoheterocyclic compounds

39	19-Nor-5-androstenediol	2.60	Lipids and lipid-like molecules
40	1-Formylneogrifolin	2.60	Lipids and lipid-like molecules
41	Floionolic acid	2.57	Lipids and lipid-like molecules
42	Porphobilinogen	2.56	Organic nitrogen compounds
43	PC(14:1(9Z)/22:2(13Z,16Z))	2.55	Lipids and lipid-like molecules
44	Oleic acid	2.51	Lipids and lipid-like molecules
45	Gymnodimine	2.47	Organoheterocyclic compounds
46	Trans-2-Hexenyl 2-methylbutyrate	2.47	Lipids and lipid-like molecules
47	Diosmetin	2.43	Phenylpropanoids and polyketides
48	(-)-Nopol	2.40	Lipids and lipid-like molecules
49	Polyoxyethylene (600) mono- ricinoleate	2.33	-

50	Oxymetholone	2.31	-
51	N-lactoyl-Methionine	2.26	Organic acids and derivatives
52	(24R)-Ergost-4-ene-3,6-dione	2.26	Lipids and lipid-like molecules
53	PS(18:0/18:1(9Z))	2.22	Lipids and lipid-like molecules
54	PE-NMe2(14:0/16:1(9Z))	2.20	Lipids and lipid-like molecules
55	5,8,12-Trihydroxy-9-octadecenoic acid	2.20	Lipids and lipid-like molecules
56	Grevilline A	2.18	Organoheterocyclic compounds
57	1-hydroxy-4-(4-hydroxy-3-methoxyphenyl)butan-2-one	2.16	Benzenoids
58	2-Pyrrolidone-5-carboxylic acid, methyl ester	2.14	-
59	()12,13-DiHOME	2.14	-

60	Angiotensin IV	2.09	Nucleosides, nucleotides, and analogues
61	PS(16:0/16:0)	2.06	Lipids and lipid-like molecules
62	PE(15:0/14:0)	2.05	Lipids and lipid-like molecules
63	2-Amino-4-[(2-hydroxy-1-oxopropyl)amino]butanoic acid	0.01	Organic acids and derivatives
64	Oryzalide B	0.05	Lipids and lipid-like molecules
65	1-Oleoylglycerophosphoserine	0.05	Lipids and lipid-like molecules
66	Mangalkanyl glucoside	0.08	Organic oxygen compounds
67	Sorbitan oleate	0.08	Lipids and lipid-like molecules
68	Janthitrem F	0.09	Organoheterocyclic compounds
69	Valyl-Tyrosine	0.10	Organic acids and derivatives

70	Asparaginyl-Phenylalanine	0.11	Organic acids and derivatives
71	Cytochalasin Opho	0.11	Alkaloids and derivatives
72	Norfloxacin	0.11	Organoheterocyclic compounds
73	Gingerglycolipid A	0.14	Lipids and lipid-like molecules
74	LysoPC(18:2(9Z,12Z))	0.16	Lipids and lipid-like molecules
75	1-(9Z,12Z,15Z-octadecatrienoyl)-glycero-3-phosphate	0.16	Lipids and lipid-like molecules
76	1-docosanoyl-glycero-3-phosphate	0.18	Lipids and lipid-like molecules
77	5'-Carboxy-gamma-chromanol	0.19	Organoheterocyclic compounds
78	Cryptomeridiol 11-rhamnoside	0.19	Lipids and lipid-like molecules
79	Ophthalmic acid	0.19	Organic acids and derivatives

80	Gamma-Glu-Leu	0.19	-
81	PGP(a-13:0/a-13:0)	0.20	Lipids and lipid-like molecules
82	MG(0:0/18:3(9Z,12Z,15Z)/0:0)	0.22	Lipids and lipid-like molecules
83	15(S)-Hydroxyeicosatrienoic acid	0.24	Lipids and lipid-like molecules
84	1-Stearoylglycerophosphoserine	0.25	Lipids and lipid-like molecules
85	Serylphenylalanine	0.00	Organic acids and derivatives
86	Palmitoyl glucuronide	0.28	Lipids and lipid-like molecules
87	HYMECROMONE METHYL ETHER	0.29	-
88	PA(16:1(9Z)/0:0)	0.30	-
89	PS(18:1(9Z)/0:0)	0.31	-
90	LysoPE(0:0/20:2(11Z,14Z))	0.32	Lipids and lipid-like molecules

91	1-Acetoxy-2-hydroxy-5,12,15-heneicosatrien-4-one	0.35	Lipids and lipid-like molecules
92	Gamma-Glutamylphenylalanine	0.35	Organic acids and derivatives
93	Crispolide	0.37	Lipids and lipid-like molecules
94	Threoninyl-Isoleucine	0.40	Organic acids and derivatives
95	Valyl-Asparagine	0.41	Organic acids and derivatives
96	ALANYL-dl-PHENYLALANINE	0.44	-
97	LysoPA(0:0/18:2(9Z,12Z))	0.46	Lipids and lipid-like molecules
98	Serylisoleucine	0.46	Organic acids and derivatives
99	1-Oleoylglycerophosphoinositol	0.48	Lipids and lipid-like molecules
100	ALANYL-dl-LEUCINE	0.48	-

101	(+)-15,16-Dihydroxyoctadecanoic acid	0.48	Lipids and lipid-like molecules
102	LysoPE(0:0/20:3(11Z,14Z,17Z))	0.48	Lipids and lipid-like molecules
103	All-trans-heptaprenyl diphosphate	0.49	Lipids and lipid-like molecules
104	Glutamylisoleucine	0.50	Organic acids and derivatives
